# Signaling Networks Regulating Metastatic Progression in Triple-Negative Breast Cancer

**DOI:** 10.3390/cells15090809

**Published:** 2026-04-29

**Authors:** Zuzanna Senkowska, Katarzyna Owczarek, Karolina Niewinna, Urszula Lewandowska

**Affiliations:** Department of Biochemistry, Faculty of Medicine, Medical University of Lodz, 92-215 Lodz, Poland; zuzanna.senkowska@umed.lodz.pl (Z.S.); katarzyna.owczarek@umed.lodz.pl (K.O.); karolina.niewinna@umed.lodz.pl (K.N.)

**Keywords:** triple-negative breast cancer, metastasis, signaling pathways, epithelial–mesenchymal transition, tumor microenvironment, cancer stem cells

## Abstract

**Highlights:**

**What are the main findings?**

- PI3K/Akt, TGF-β, Wnt/β-catenin, NF-κB, and Rho/ROCK pathways coordinately regulate key steps of the metastatic cascade in TNBC.
- TNBC molecular heterogeneity is linked to differential activation of signaling networks driving EMT, invasion, and metastatic colonization.

**What are the implications of the main findings?**

- Mapping pathway activity to specific metastatic stages provides a mechanistic framework for interpreting TNBC progression.
- Targeting signaling dependencies associated with TNBC subtypes may support the development of more precise therapeutic strategies.

**Abstract:**

Triple-negative breast cancer (TNBC) is one of the most aggressive and clinically challenging subtypes of breast cancer, defined by the absence of estrogen receptor, progesterone receptor, and human epidermal growth factor receptor 2 expression. The lack of actionable molecular targets contributes to limited therapeutic options, frequent recurrence, and a high propensity for distant metastasis. Metastatic dissemination remains the principal cause of mortality in patients with TNBC and is driven by complex molecular mechanisms involving multiple interconnected signaling networks. This review summarizes current knowledge of the molecular mechanisms underlying metastatic progression in TNBC, with particular emphasis on signaling pathways that regulate tumor invasion, migration, and colonization of distant organs. We discuss the roles of key pathways, including PI3K/Akt, TGF-β, Wnt/β-catenin, NF-κB, and Rho/ROCK signaling, in the regulation of epithelial–mesenchymal transition, cytoskeletal remodeling, cancer stem cell phenotypes, and tumor–microenvironment interactions. A deeper understanding of these signaling networks may facilitate the identification of novel therapeutic targets and support the development of more effective strategies to limit metastatic disease in TNBC.

## 1. Introduction

Breast cancer (BC) is one of the most prevalent malignancies and represents the fifth leading cause of cancer-related deaths [1]. Based on data from 185 countries, it is estimated that in 2022, 2.3 million new BC cases were diagnosed worldwide, and 670,000 deaths were reported among women [2]. Although it is a disease that mainly impacts the female gender, it is worth noting that approximately 1% of all BC cases are diagnosed in men [3,4]. This disease affects women of all ages; however, the likelihood increases with age. It was once mostly reported in developed countries, but with the development of and wider access to advanced diagnostic methods, in 2020 half of diagnoses and two-thirds of related deaths occurred in less developed countries. Apparently, BC incidence is associated with human development. This is measured using the Human Development Index (HDI), an index integrating life expectancy, educational attainment, and economic indicators. HDI provides a more comprehensive and effective comparison between countries than income alone. Data on BC incidence, standardized by women’s age and ethnicity, indicate that 48 per 100,000 individuals are affected. Substantially lower rates are noted among the populations of sub-Saharan Africa, where it is significantly below average, at less than 30 per 100,000. In contrast, countries in Western Europe and North America exhibit markedly higher incidence levels, exceeding 70 per 100,000. Another indicator that perhaps even further highlights differences between countries is the Mortality-to-Incidence Ratio (MIR). Africa’s MIR of 0.460 reflects substantial systemic limitations in cancer detection, treatment, and overall healthcare capacity. In contrast, Northern America (MIR = 0.162) exhibits much lower MIRs globally, indicative of well-developed healthcare facilities and more effective cancer management [5,6]. It is projected that if the current pattern of yearly increases in the number of BC diagnoses and mortality persists, by 2040, considering solely demographic factors such as population growth and aging, over 3 million new cases and 1 million deaths per year will be observed [7]. These rising numbers highlight the urgent need to enhance cancer awareness, improve screening programs, expand access to advanced diagnostic modalities, and provide effective personalized treatments. In fact, it should also be emphasized that the main determinant of mortality in BC is related to the occurrence of metastases. Complications resulting from recurrent or metastatic disease account for approximately 90% of patient deaths [8,9,10].

In the past several decades, cancer therapy has evolved from using nonspecific cytotoxic agents to the development of targeted cancer therapies. Surgery has been the first successful treatment for cancer. Surgical techniques have benefited from increased understanding of the cancer process, advancement in imaging technology, and technological improvements in surgical equipment. Early detection of cancers is another factor that has contributed to the success of surgery. Nonetheless, it is associated with many risks, including mortality and morbidity, which are dependent upon the type of surgery involved [11]. Radiotherapy is the second main type of standard cancer treatment and is used in about half of all patients. It works by using high-energy radiation to destroy cancer cells. Today, doctors try to protect healthy tissues by using advanced methods such as four-dimensional computed tomography, which helps map the tumor very precisely but requires the patient to be positioned and kept still during treatment. Radiotherapy is often combined with surgery to shrink tumors before an operation and to remove any tiny cancer cells that may remain afterward [12]. The last but not least important cancer management strategy is chemotherapy, meaning a drug treatment. It is most lethal to fast-growing cells, which means that besides targeting cancer cells it has many side effects which are especially noticeable in normal cells with high proliferation, such as hematopoietic cells, upper and lower gastrointestinal tract cells, and hair follicles [11]. Recent breast-cancer-focused reviews emphasize that modern care increasingly integrates genomic profiling, targeted agents, and immunotherapy, contributing to declining mortality despite rising incidence [13]. Patients with triple-negative breast cancer (TNBC) do not respond to hormone therapy or human epidermal growth factor receptor 2 (HER2)-targeted drugs because they lack the necessary receptors. For this reason, standard treatment for TNBC that cannot be treated with surgery is still general chemotherapy. This subtype often responds well to common chemotherapy drugs such as taxanes and anthracyclines. Some small-molecule drugs, including bromodomain and extra-terminal domain inhibitors, have shown promising results, but cancer cells quickly develop resistance through different mechanisms. Therefore, it is necessary to better understand the molecular features of TNBC, target changes within the tumor and its surrounding environment, and develop new treatment strategies. Because TNBC is aggressive, diverse, and prone to drug resistance, combining multiple targeted therapies is essential to improve patient outcomes [14]. In this dynamic environment, the signaling pathways have come to represent the link between cancer biology and clinical practice. The signaling network involved in breast cancer progression and metastasis comprises PI3K/Akt, Rho/ROCK, TGF-β, Wnt, and NF-κB signaling pathways, which play an integral role in cell proliferation, survival, epithelial–mesenchymal transition, immune escape, and metastasis formation. Pathway knowledge has led to a paradigm shift in drug development, resulting in a range of drugs, including PI3K inhibitors, CDK4/6 inhibitors, PARP inhibitors, and immune checkpoint therapies, which have become a cornerstone of breast cancer treatment in advanced stages. Recent evidence shows that the crosstalk between signaling pathways leads to therapy resistance and recurrence of metastases [15,16,17]. These advances show that signal transduction mechanisms are not just descriptive elements, but crucial to therapy, drug resistance, and metastatic control. The aim of this review is to expand the understanding of the metastatic properties of BC, particularly the triple-negative subtype, and to describe the molecular processes that influence the development of distant metastases.

## 2. Heterogeneity and Subtypes of Breast Cancer

Based on morphological differences, approximately 20 subtypes can be distinguished among BC, making it a very heterogeneous disease. This is mostly due to discrepancies in etiology and pathological characteristics. Furthermore, some cancers grow rapidly, making them clinically very aggressive, whereas those that develop slowly offer a promising prognosis for recovery and longer survival [18,19]. Not only does heterogeneity occur between different patients or metastases (called intertumor heterogeneity), but also within the individual tumor from the same patient (called intratumor heterogeneity). Inter-patient variability reflects differences in genetic background, tumor biology and clinical behavior across individuals. It is especially noticeable among TNBC patients who exhibit distinct molecular subtypes with plenty of genetic, epigenetic, transcriptomic and proteomic variations, metabolic dependencies, and dominant signaling pathway activation. This results in differences in metastatic patterns and overall prognoses. In particular, variation in germline BRCA mutation and programmed cell death ligand 1 (PD-L1) is particularly important when selecting treatment for patients with TNBC. Due to very high genomic instability and increased immune infiltration, some patients may have significantly elevated PD-L1 expression compared to other subtypes. However, in the case of BRCA, BRCA1 usually has higher expression, meaning cells cannot repair double-strand breaks, which causes cell death. As a result, patients with apparently similar clinicopathological features may experience markedly different outcomes. Recognizing and integrating inter-patient variability is therefore essential for accurate risk stratification, biomarker development, and the implementation of personalized therapeutic strategies in TNBC [20,21].

**Figure 1:**
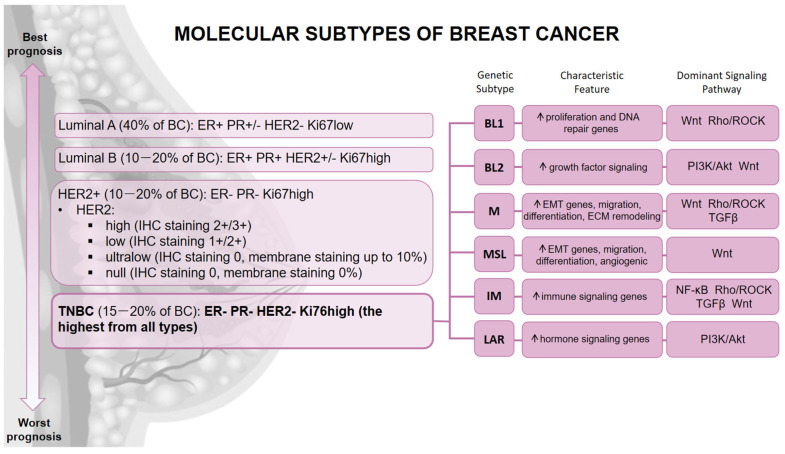
Molecular classification of breast cancer and TNBC heterogeneity. Breast cancer is classified into several molecular subtypes based on the expression of hormone receptors, human epidermal growth factor receptor 2 (HER2), and the proliferation marker Ki-67. The major subtypes include luminal A, luminal B, HER2-positive, and triple-negative breast cancer (TNBC). Luminal A tumors are characterized by ER and PR positivity, lack of HER2 expression, and low proliferative activity, and are associated with the most favorable prognosis. Luminal B tumors also express hormone receptors but exhibit higher Ki-67 levels and may show HER2 expression, resulting in a more aggressive phenotype. HER2-positive tumors lack hormone receptor expression but demonstrate HER2 overexpression and high proliferative activity. TNBC is defined by the absence of ER, PR, and HER2 expression and is associated with the highest Ki-67 levels and the poorest prognosis. TNBC can be further subdivided into several molecular subtypes, including basal-like (BL1 and BL2), mesenchymal (M), mesenchymal stem-like (MSL), immunomodulatory (IM), and luminal androgen receptor (LAR), which differ in gene expression profiles and biological behavior. The figure also illustrates how TNBC heterogeneity translates into differential pathway usage, highlighting the dominant signaling dependencies associated with each TNBC subtype. Arrow symbols indicate directional biological relationships: “↑” indicates increased activity or outcome.

According to cancer cells’ ability to infiltrate into normal tissue and to form metastases, BC is divided into non-invasive and invasive subtypes. Among invasive subtypes based on an anatomical structure that has been affected, invasive ductal carcinoma (IDC) and invasive lobular carcinoma (ILC) can be distinguished. In addition to the histological division, BC is also classified according to the molecular subtype based on the expression of specific receptors and the Ki-67 protein [22,23]. The Ki-67 is a marker protein utilized to distinguish proliferating cells [24]. Staining pattern analysis indicated that it is present in the nuclei of cells progressing through the G1, S, and G2 phases, as well as during mitosis. On the contrary, quiescent cells in the G0 phase lack Ki-67 expression [25]. The localization of Ki-67 also depends on the phase of the cell cycle. During the interphase, Ki-67 can only be detected within cell nuclei, while in mitosis, most of the protein is displaced to the surface of cellular chromosomes. Due to the reliable correlation of Ki-67 with the proliferative activity of cancer cells, immunohistochemical (IHC) analysis for the presence or absence of this protein is now essential for defining several cancer types [24,26,27]. Together with determining Ki-67 expression, the IHC panel, which efficiently and significantly stratifies BC molecular subtypes, also includes an expression assessment of three other markers: estrogen receptor (ER), progesterone receptor (PR), and HER2. The presence, combination, or absence of these markers defines the major invasive breast cancer subtypes: luminal A, luminal B, HER2-positive, and TNBC, which are summarized in Figure 1. Several other less common subtypes can also be distinguished, such as medullary, tubular, metaplastic, and mucinous carcinomas [22,23,28].

However, the increasing demand for improved risk stratification and more precise prognostic assessment, together with a deeper understanding of tumor biology, has driven the development of multiple multigene profiling assays, including Oncotype DX [29], Prosigna (PAM50) [30], MammaPrint [31], and BluePrint [32]. The 70-gene MammaPrint signature is applied in patients with early-stage, ER-positive breast cancer to identify those at the highest risk of recurrence and most likely to benefit from the adjuvant chemotherapy. The PAM50 assay serves as a molecular classifier of BC subtypes and additionally provides an estimate of distant recurrence risk, as well as the potential responsiveness to the neoadjuvant chemotherapy [33,34,35].

### 2.1. Luminal A

The most frequently diagnosed BC subtype, accounting for approximately 40% of detected BC cases and associated with early-stage presentation, is the luminal A subtype [23]. It is characterized by overexpression of HR (hormone receptor; ER ≥ 1% with/without PR ≥ 20%/<20%), the absence of HER2 expression (≤10%), and the low expression of the cell proliferation marker Ki-67 (<14%), which causes it to be referred to as HR+, HER2−, and Ki-67 low [36,37]. Estrogen receptors (ERs; ERα and ERβ) are included in the group of intracellular receptors that mediate estrogen-dependent signaling and differentially regulate gene transcription, functioning as ligand-activated transcription factors. Through these mechanisms, they influence a wide range of cellular processes, including growth, proliferation, and differentiation [38,39]. PR works similarly, as a member of the nuclear receptor family of ligand-dependent transcription factors. It is prevalently responsible for regulating the network of target gene expression in response to binding its related steroid hormone, progesterone. PR regulates the expression of gene networks to control development, differentiation, and proliferation of target tissues [40,41]. Clinically, luminal A tumors are well-differentiated, exhibit regulated cell proliferation, and are associated with a low histological grade and favorable prognosis. They are also characterized by a reduced risk of recurrence and higher overall survival rates [23,36,37]. Beyond that these tumor type exhibit high expression of cytokeratins 7/8/18/19, which is common among luminal epithelial cells of the breast [28].

### 2.2. Luminal B

A less common type of BC, accounting for 10–20% of cases, is the luminal B subtype. Similarly to luminal A, it is also associated with early-stage presentation. Cancer cells exhibit high HR (ER+ and PR+) expression and are rapidly dividing, as indicated by high Ki-67 levels (>14%). Interestingly, HER2 expression in the luminal B subtype is not uniform, and can be either HER2+ (>10%) or HER2− (≤10%). Collectively, these markers enable to define this cancer as HR+/HER2+ and HR+/HER2−, Ki-67high. On the clinical level, this subtype has a significantly poorer prognosis than the luminal A subtype because it grows and divides more rapidly. Ki-67 expression acts as a marker to differentiate among luminal BC subtypes and to evaluate the proliferative activity and aggressiveness of tumors [23,31,42].

### 2.3. HER2-Positive

The HER2-positive (HER2+) subtype occurs at a similar frequency to luminal B, accounting for approximately 10–15% of BC cases. It is defined as HR-, HER2+, due to the absence of both ER (<1%) and PR (<20%) and overexpression/amplification of HER2 (>10%). Expression of the Ki-67 marker (>20%) indicates its highly proliferative properties [23]. The HER2 protein (also referred to as HER2/neu or ErbB2) is a cell-surface receptor belonging to the family of receptor tyrosine kinases (RTKs) encoded by the HER2 gene. It controls the transduction of extracellular signals into intracellular responses. In normal tissues, HER2 is expressed at low levels, contributing to the regulation of cell growth and division. Gene amplification can result in HER2 overexpression, leading to accelerated cellular proliferation. Upon activation, HER2 induces phosphorylation of multiple tyrosine residues at its C-terminal domain. Activated RTKs are critical for intercellular signaling and regulate diverse biological processes, including cell proliferation, motility, differentiation, and metabolism, through downstream pathways such as PI3K/Akt, Raf/MEK/MAPK, JAK/STAT, Src, and PLCγ [43,44,45]. As research progresses, a new, more precise classification of HER2+ BC has emerged to better identify patients: HER2-low, HER2-ultralow, and HER2-null subtypes. Unlike standard HER2+ BC, these subtypes have low levels of HER2 protein expression. The tumor is categorized as HER2-null, indicating that it is unlikely to benefit from HER2-targeted therapies when the IHC result is 0 with an absence of membranous staining. When the result is 0 and accompanied by faint membranous staining in up to 10% of tumor cells, then it is designated HER2-ultralow, a subgroup that may still exhibit sensitivity to antibody–drug conjugates (ADCs). Tumors scoring IHC1+ or IHC2+ with negative HER2 fluorescence in situ hybridization (FISH) are classified as HER2-low, and these lesions can also respond to ADC-based treatments. In contrast, cases with IHC2+ and positive FISH result or IHC3+ are defined as HER2+, for which HER2-directed therapies are the standard of care. HER2+ BCs are typically aggressive and associated with poor clinical outcomes, however, the introduction of HER2-targeted therapeutic agents has substantially improved patient prognosis [23,46,47,48,49].

### 2.4. Triple Negative

This subtype accounts for approximately 15–20% of all BC cases. It is characterized by the lack of expression of all three receptors (ER < 1%, PR < 20%, and HER2 ≤ 10%), which is why it is also called HR−/HER2−. This makes hormonal and targeted therapies ineffective. It is the most aggressive subtype with the highest proliferation index of Ki-67 (>30%), which gives it the worst prognosis [23,36]. It is also a very heterogeneous subtype. On the basis of its gene expression profile, it can be further classified into basal-like 1 (BL1), and 2 (BL2), mesenchymal (M), mesenchymal stem-like (MSL), immunomodulatory (IM), and luminal androgen receptor (LAR) subtypes [50]. The BL1 and BL2 subtypes exhibit sensitivity to DNA-damaging chemotherapeutic agents, such as cisplatin, and are marked by increased expression of genes involved in cell cycle regulation and DNA damage responses. The BL1 subtype is distinguished by particularly high levels of cell proliferation and DNA repair-related transcripts, as well as elevated Ki-67. In contrast, the BL2 subtype is characterized by enhanced activity of growth factor signaling pathways, elevated glycolytic and gluconeogenic processes, and increased expression of myoepithelial-associated markers (P-cadherin). The M and MSL subtypes are associated with reduced distant metastasis-free survival and demonstrate responsiveness to inhibitors of the phosphoinositide 3-kinase (PI3K)/mechanistic target of rapamycin (mTOR) pathway. Their transcriptional profiles share considerable similarities with those of chemoresistant metaplastic BC and show enhanced expression of genes involved in epithelial–mesenchymal transition (EMT), cell migration, extracellular matrix remodeling, and cellular differentiation. While the M subtype is distinguished by elevated expression of proliferation-related genes, the MSL subtype is enriched for mesenchymal stem cell-associated transcripts and exhibits increased expression of angiogenic and growth factor-mediated signaling genes. Additionally, the MSL subtype is characterized by reduced expression of claudins 3, 4, and 7. Increased expression of immune signaling genes (immune cell and cytokine signaling, antigen processing and presentation, core immune signaling pathways) is the main characteristic of the IM subtype. Both of these subtypes show a relatively good prognosis. The expression profile of the IM subtype is generated predominantly by tumor-infiltrating lymphocytes (TILs) rather than tumor cells themselves. Approximately 20% of TNBCs show a substantial presence of TILs. This was found to be an independent prognostic marker in TNBC. The presence of TILs correlates with improved responses to both adjuvant and neoadjuvant treatments and may function as an indicator of more favorable prognosis when identified in residual disease following neoadjuvant therapy. Last but not least is the LAR subtype, which is characterized by enrichment in genes responsible for hormone signaling, steroid synthesis, and androgen/estrogen metabolism including overexpression of AR and its downstream targets and coactivators. Individuals with the LAR subtype exhibit reduced relapse-free survival [51,52,53,54]. Furthermore, detailed genomic analysis performed by Bareche et al. revealed genomic alterations characteristic for each TNBC molecular subtype. BL1 tumors arise to have high levels of chromosomal instability, high rate of TP53 mutations (92%), copy-number gains and amplifications of PI3KCA and AKT2, accompanied by deletions in genes involved in DNA repair mechanisms. In contrast, the LAR subtype is characterized by higher mutational burden and enrichment in mutations of PI3KCA, AKT1 and CDH1 genes. Both M and MSL subtypes are associated with higher signature that predispose to angiogenesis. As anticipated, high expression levels of immune response-associated signatures and checkpoint inhibitor genes, including cytotoxic T-lymphocyte-associated antigen-4, programmed cell death protein-1, and PD-ligand 1 are present in the IM group. This pattern should be related to the contamination by the immune infiltrate. Notably, the LAR subtype was linked to poorer prognosis, whereas the IM subtype was associated with more favorable clinical outcomes [50,52,55]. Importantly, these molecular subtypes are characterized not only by distinct gene expression profiles but also by differential activation of specific signaling pathways that drive metastatic behavior. For example, mesenchymal (M) and mesenchymal stem-like (MSL) subtypes are enriched in epithelial–mesenchymal transition, extracellular matrix remodeling, and increased activity of pathways such as TGF-β, Wnt/β-catenin, and PI3K/Akt, which are associated with enhanced invasive and migratory capacity [56,57]. In contrast, the immunomodulatory (IM) subtype is characterized by elevated immune-related signaling, including cytokine and NF-κB pathway activation, reflecting the strong contribution of tumor-infiltrating lymphocytes [57]. The luminal androgen receptor (LAR) subtype is defined by androgen receptor signaling and metabolic pathway dependencies, which may influence tumor growth and therapeutic responsiveness [58]. Basal-like subtypes (BL1 and BL2) are associated with increased proliferation and DNA damage response signaling, including PI3K/Akt pathway activity [59]. These subtype-specific signaling patterns highlight the functional heterogeneity of TNBC and provide a mechanistic basis for the differential metastatic behavior illustrated in Figure 1. Moreover, inter-patient variability and germline genetic background not only contribute to differences in tumor incidence, but also functionally influence signaling pathway activity and therapeutic response [20,21]. Even though most TNBCs have dysregulated signaling pathways such as RHO/ROCK, PI3K/Akt, Wnt, TGFbeta, NFkB, the molecular drivers of activation differ widely between patients, which causes different downstream effects [60]. The PI3K/AKT pathway is activated in most cases of TNBC through a variety of mechanisms, including PIK3CA mutation, PTEN loss, receptor tyrosine kinase amplification, and non-genetic or microenvironmental signaling inputs. This leads to a wide range of downstream effects, from proliferative and metabolic reprogramming to survival signaling, therapy resistance, and metastatic competence [61]. Wnt/β-catenin signaling also exhibits significant functional heterogeneity in TNBC tumors, characterized by varied activation through ligand overexpression, epigenetic silencing of pathway inhibitors, or stromal Wnt secretion. This variability leads to variations in the maintenance of cancer stem cells, EMT, and metastatic relapse, often working with PI3K signaling to stabilize β-catenin and boost invasion and tumor-initiating ability [62]. TGF-β signaling is highly context dependent, influenced by treatment exposure and tumor–stroma interactions. Chemotherapy has been demonstrated to enhance TGF-β signaling in specific subsets of TNBC, facilitating cancer stem cell proliferation, EMT, metastatic re-initiation, and resistance to cytotoxic and immune-based therapies [63]. Differences between patients in the composition of the extracellular matrix, the number of stromal cells, and the mechanical properties of the tumor microenvironment (TME) also affect RHO/ROCK signaling. This leads to differences in cytoskeletal remodeling, invasion plasticity, and intravasation efficiency [64]. At the same time, differences in inflammatory and cytokine signaling lead to different levels of NF-κB pathway activation. This pathway combines signals from the tumor itself and its surroundings to help the tumor avoid the immune system, grow new blood vessels, and become resistant to treatment [65].

Heterogeneity of both molecular and functional aspects in TNBC leads to different parallel pathways for metastatic spread and at the same time produces subtype-specific susceptibilities to treatments. Different mechanisms of survival, interaction with immune cells and growth at a metastatic site define the type of signaling pathway involved. The combination of mutations in PIK3CA along with deletion of CDKN2A and retention of RB1 are commonly seen in the LAR type of TNBC, making it more sensitive to PI3K-AKT and CDK4/6 inhibitors [66]. At the same time, variations in autophagy and DNA damage repair contribute to therapy-induced immunogenicity, where certain TNBC models show type I interferon response post irradiation. At the same time others depend on autophagy suppression to induce an NF-kB-mediated immune response, and PD-L1 expression post-radiation emphasizes the necessity of a combinatorial approach with an immune checkpoint blockade [67]. Immune and metabolic stratifications also reveal the difference, where the former is expected to respond better to immunotherapy and RAS/WNT inhibitors, while the latter will be sensitive to PI3K inhibition and anti-angiogenic therapies [68].

This indicates that patient characteristics influence the signaling pathways, thereby establishing individualized states of signaling networks leading to metastasis and treatment variability. Therefore, stratifying based on the signaling pathways is essential when considering TNBC treatment options.

## 3. Metastasis

One of the most important hallmarks of cancer is its ability to metastasize. This feature is responsible for the greatest number of cancer-related deaths [69,70]. Metastasis refers to the formation of secondary malignant lesions at a part of the body distant from the primary tumor. This process comprises a cascade of biological steps in which cancer cells gradually acquire the ability to invade through the mucosal barrier, infiltrate deeper tissues, and subsequently disseminate via the bloodstream, lymphatic system, or direct extension into close structures. These disseminated cells then seed remote organs and eventually regain proliferative capacity to establish metastatic lesions. Each stage of this progression is facilitated by the tumor cells’ capacity to switch between distinct phenotypic states and to manipulate surrounding immune and stromal components within the TME, enabling their survival, expansion, and evasion of immune surveillance [70,71,72].

Development of distant metastasis begins with the entry of primary tumor cells into the surrounding stromal compartment through migratory and invasive activity [73], which should be mechanistically distinguished from EMT, a phenotypic reprogramming process that enables, but does not itself constitute, invasion. EMT is governed by transcription factors including Twist, Snail, Slug, and FOXC2, which induce a shift toward a mesenchymal-like phenotype characterized by decreased E-cadherin expression and increased expression of N-cadherin, fibronectin, and vimentin, thereby reducing cell–cell adhesion and increasing cellular plasticity. The invasion process requires disruption of the basement membrane that surrounds the primary lesion. This stage is mechanistically defined by active migration and extracellular matrix (ECM) remodeling, including proteolytic degradation mediated by matrix metalloproteinases (MMPs) and cytoskeletal reorganization regulated by pathways such as Rho/ROCK. Tumor cells can detach from the main mass either individually or as multicellular groups [74]. During collective invasion, clusters of cells undergo EMT, move away from the primary tumor, and penetrate the basement membrane. Importantly, these cells often retain partial epithelial characteristics, adopting a hybrid EMT phenotype that preserves intercellular adhesion while enabling coordinated invasion. Movement of these clusters is governed by specialized leader cells that guide other cells [75]. In contrast, single-cell invasion relies on autonomous motility programs, including amoeboid or mesenchymal migration, and is generally less efficient due to the lack of cooperative interactions observed in collective invasion. Single cells that survive either look for pre-existing gaps within the extracellular matrix (ECM) or secrete MMPs to degrade or remodel the ECM, further supporting their invasive potential [76].

Intravasation, the subsequent stage of the metastatic cascade, is facilitated by the “leakiness” of the neo-angiogenic and irregular blood vessels, which are more cell permeable [77]. Importantly, intravasation represents a biologically distinct step from invasion, as it depends on tumor–microenvironment interactions and vascular remodeling rather than ECM degradation alone. This pathological angiogenesis results from inflammatory cytokines and growth factors present in the TME. Moreover, the hypoxic conditions there stimulate neoplastic cells to secrete pro-angiogenic factors, such as vascular endothelial growth factor (VEGF), fibroblast growth factor (FGF), platelet-derived growth factor (PDGF), angiopoietins, hepatocyte growth factor (HGF), transforming growth factor-β (TGF-β), and matrix metalloproteinases (MMPs). Tumor cells can enter the vascular endothelium and circulation either by disrupting endothelial junctions or through amoeboid invasion [78]. This process is strongly supported by tumor-associated macrophages (TAMs), which are not essential for earlier invasion steps but play a critical role during intravasation [79]. TAMs secrete multiple factors including epidermal growth factor (EGF) and attract EGFR-expressing tumor cells to the perivascular region while tumor cells in turn express colony stimulating factor 1 (CSF1) and attract more TAMs, resulting in a positive feedback loop creation. As a consequence, the combination of endothelial cells, TAMs, and tumor cells organize the TME of metastasis (TMEM). A subset of TAMs located within perivascular TMEM structures expresses high levels of the angiopoietin-1 receptor TIE2 (tyrosine kinase with immunoglobulin-like and EGF-like domains). These TIE2-positive TAMs release VEGFA, inducing short-lived increases in local vascular permeability that, in turn, further promote tumor cell invasion [76].

The process of cancer cells entering the lumen of a blood vessel is very demanding and only a few cancer cells reach the circulation. However, survival of these cells is even harder with the hemodynamic shear forces, immune stresses, and red blood cell collisions they encounter once there [80]. Extravasation, in contrast to intravasation, requires firm adhesion to the endothelium and active transendothelial migration rather than passive entry through permeable vessels. This stage introduces distinct selective pressures compared to earlier steps, including resistance to anoikis, immune evasion, and adaptation to hemodynamic shear stress conditions in circulation. This stage is therefore mechanistically distinct from earlier steps such as EMT and invasion, as it primarily depends on survival under circulatory stress rather than migratory capacity. Circulating tumor cells (CTCs) arrest in a vessel and extravasate through two primary mechanisms: physical occlusion and adhesion after rolling. In the physical occlusion mechanism, the diameter of a CTC exceeds that of the microvessel, causing the cell to become mechanically trapped, after which it can adhere to the vessel wall and subsequently extravasate. In the rolling-adhesion mechanism, CTCs first make contact with the endothelium, then roll along its surface through interactions mediated by E-selectin or P-selectin, eventually being stopped through binding to αvβ3 integrin, intercellular adhesion molecule-1 (ICAM-1) or vascular cell adhesion molecule-1 (VCAM-1) [72,81].

Following extravasation, cancer cells must accomplish a final step which is establishing growth within the secondary organ. This colonization phase is considered highly inefficient, as only a small fraction of CTCs successfully adapts to the foreign microenvironment, evades immune surveillance, and reinitiates proliferative growth to form macroscopic metastatic lesions [82]. Successful colonization depends on the ability of tumor cells to interact with the pre-metastatic niche, engage local stromal components, and activate survival and growth signaling pathways.

A pre-metastatic niche (PMN), created by the initial tumor in distant organs, is also essential to the metastasis process since it facilitates the previously mentioned steps [83]. Once PMN is established, the local microenvironment becomes recognized by immunosuppressive conditions, increased vascular permeability, and elevated angiogenic activity, each supporting the successful seeding and expansion of incoming tumor cells. More than a century ago, Paget first mentioned the “seed and soil hypothesis”, and since then, researchers have been exploring the mechanisms of cancer metastasis. It stated that the spread of tumors relies on the interaction and cooperation between the cancer cells (seed) and the host organ (soil). Subsequently, Lyden was the first to introduce the concept of the PMN. Recent studies increasingly demonstrate that tumors can prepare distant sites by forming PMN before metastatic cells arrive, effectively creating a microenvironment that favors their eventual colonization and growth [84,85]. These late stages of metastasis, particularly colonization, are strongly influenced by both tumor-intrinsic properties and the compatibility with the microenvironment of target organs, a phenomenon referred to as organotropism. This phenomenon has been extensively investigated in BC, where distinct molecular subtypes exhibit preferential metastasis to specific organs [70].

These mechanistic determinants of metastasis translate into clinically observable patterns of organ-specific dissemination in breast cancer. In addition to regional relapse, breast cancer preferentially metastasizes to specific distant organs, including bones [86], brain [87], liver [88], lungs [89], and lymph nodes [90]. Bones represent the predominant site of distant metastasis, occurring in approximately 70% of patients with metastatic BC. The liver is the next most frequent metastatic region, affecting roughly 30% of cases, followed by the brain, which is involved in about 10–30% of patients. Notably, BC subtypes differ markedly in both overall survival outcomes and in their tendencies to metastasize to particular organs [8]. These organ-specific patterns are driven by both tumor-intrinsic signaling programs and interactions with the local microenvironment, including chemokine gradients, adhesion molecules, and niche-specific stromal support. A study conducted in 2015, which included 531 individuals with associated distant (bones, visceral organs, or brain) metastasis, revealed a correlation between BC subtype and metastatic behavior. The skeleton was the most common site of relapse, representing approximately half (48%) of the patients, followed by liver (27%), lungs (23%), central nervous system (CNS; 17%), and pleura (7%). Bone metastasis was significantly associated with the luminal A and B subtypes. Liver relapse was commonly detected in the HER2 subtype, while in patients with TNBC, pleura and lung metastasis mostly occurred. Furthermore, both the HER2 subtype and TNBC were significantly associated with CNS relapse [91]. A later retrospective, population-based cohort study conducted on bigger scale (over 240,000 patients, including over 220,000 cases in control groups and almost 17,500 cases with distant metastasis) showed similar patterns. Consistently, breast cancer subtypes exhibited distinct site-specific metastatic patterns. Across all BC subtypes, bones were the most common site of relapse. Patients with the HR−/HER2+ subtype exhibited an increased likelihood of developing brain metastases. Additionally, liver metastases were more frequently observed in HER2-positive subtypes compared with HER2-negative subtypes, whereas patients with TNBC predominantly developed lung metastases [92].

## 4. TNBC Metastasis

**Figure 2:**
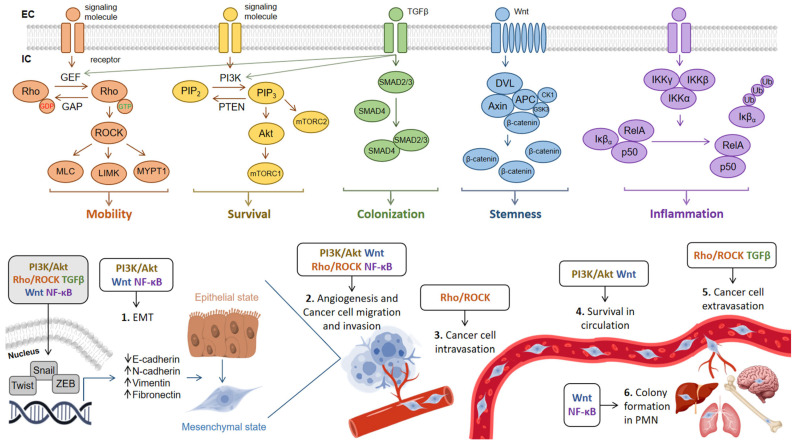
Signaling pathways regulating metastatic progression in triple-negative breast cancer (TNBC). Key pathways (PI3K/Akt, TGF-β, Wnt/β-catenin, NF-κB, and Rho/ROCK) are mapped to distinct stages of the metastatic cascade, including epithelial–mesenchymal transition (EMT), migration and invasion, intravasation, survival in circulation, extravasation, and colonization in the pre-metastatic niche (PMN). Functional annotations indicate causal relationships between pathway activation and phenotypic outcomes, such as EMT induction (E-cadherin, vimentin, fibronectin), cytoskeletal remodeling, enhanced cell motility, survival under stress conditions (including anoikis resistance), immune evasion, and metastatic colonization. Arrow symbols indicate directional biological relationships: “→” denotes a sequential or causal effect, “↑” indicates increased activity or outcome, and “↓” indicates decreased activity or outcome.

In contrast to HR+ or HER2+ subtypes, TNBC is known for its aggressiveness with larger tumor size, lymph node involvement, higher tumor grade, higher conversion rate from localized TNBC to metastatic TNBC, and a tendency to affect younger patients [93]. Lungs are one of the most prevalent sites of distant metastasis in TNBC, accounting for 40% of the cases of metastasis [94,95]. In BC patients whose metastases are limited to the lungs, the median survival is reported to be 25 months; however, for the TNBC subtype, this is considerably shorter, with a median survival of only 11 months [96]. There are many signaling pathways, proteins, and molecules that predispose TNBC to its highly aggressive phenotype. The pathological upregulation of signaling pathways leads to tumor recurrence and metastasis, which are immediate causes of the poor prognosis of TNBC. The key signaling pathways regulating epithelial–mesenchymal transition, tumor cell migration, invasion, and metastatic colonization in TNBC are summarized in Figure 2. Importantly, individual signaling pathways do not act uniformly across the metastatic cascade but instead regulate specific stages of this process. For instance, TGF-β and Wnt/β-catenin signaling are key drivers of EMT and the acquisition of invasive phenotypes, whereas Rho/ROCK signaling predominantly regulates cytoskeletal dynamics and cell motility during invasion and intravasation. PI3K/Akt signaling supports cell survival and metabolic adaptation, particularly in circulating tumor cells, while NF-κB contributes to inflammatory signaling, immune evasion, and metastatic colonization. This stage-specific activity highlights the importance of context-dependent pathway activation in metastatic progression. Beyond their stage-specific roles in TNBC metastasis, these signaling pathways are also broadly implicated in tumor progression across multiple cancer types. The Rho/ROCK pathway induces cytoskeletal dynamics and cell contractility, enhancing the invasive behavior of prostate, liver, and colorectal cancers [97]. PI3K/Akt signaling drives proliferation and cell survival across a broad spectrum of solid tumors [98], while TGFβ acts as a regulator of EMT and invasion in pancreatic and colorectal cancers [99]. Wnt signaling promotes stemness and metastatic colonization in colorectal and gastric cancers [100], whereas NF-κB mediates inflammatory and survival programs that facilitate metastasis in colorectal, ovarian, melanoma, and hepatocellular carcinomas [101]. Consistent with these observations, experimental studies in colorectal and hepatocellular carcinoma models have demonstrated that PI3K/Akt activation enhances tumor cell survival, migration, and metastatic potential, as evidenced by in vitro invasion assays and in vivo metastasis models. Similarly, NF-κB-driven signaling has been shown to promote inflammatory microenvironment remodeling, immune evasion, and metastatic dissemination across multiple cancer types, supported by both preclinical and clinical studies.

### 4.1. Rho/ROCK Signaling Pathway

The Rho/ROCK signaling pathway is responsible for a broad range of physiological and pathological processes among which actin cytoskeletal dynamics modulation seems the most crucial [97,102]. In the process of carcinogenesis, this makes it play a key role in invasion, intravasation and extravasation. During transcellular transmigration, breast cancer cells form filopodia-like protrusions that extend into the endothelium; this leads to the incorporation of CTCs into the endothelium of distant organs. This signaling pathway is also involved in cell–cell adhesion reduction and results in local disruption of the endothelial barrier [64]. It encompasses two key components, namely the Rho family of small GTPases and their major downstream effector kinases, Rho-associated coiled-coil-containing protein kinases 1 and 2 (ROCK1 and ROCK2) [97,102]. The most common and extensively studied Rho GTPases include Rac1, Cdc42, and RhoA. The activity of Rho GTPases is regulated by three groups of proteins: Rho guanine nucleotide exchange factors (RhoGEF); Rho GTPase-activating proteins (RhoGAP); and Rho GDP-dissociation inhibitors (RhoGDI) [103,104]. During the process of carcinogenesis, this signaling pathway is involved in the regulation of tumorigenicity, tumor growth, metastasis, angiogenesis, tumor cell death, and chemoresistance [105]. RhoA activation occurs under the influence of many external factors including growth factors, cytokines, thrombin, and mechanical stress, which transmit the signal through membrane receptors such as G protein-coupled receptors (GPCRs), RTKs, and integrins [106]. Following these interactions, specific GEFs are triggered to catalyze the exchange of GDP for GTP on RhoA. While activated, GTP-bound RhoA interacts directly with the Rho-binding domain of ROCK, triggering a conformational shift that disrupts its autoinhibitory interaction and activates the kinase domain. This activation allows ROCK to phosphorylate multiple downstream targets, including myosin light chain (MLC), LIM domain kinase, and the myosin phosphatase targeting subunit (MYPT1), thereby facilitating actomyosin contractility and the assembly of stress fibers. Apart from RhoA-mediated activation, ROCK1 and ROCK2 can also be induced independently by proteolytic cleavage. ROCK1 is processed by caspase-3 during apoptosis or under disease conditions, while ROCK2 is processed by granzyme B, released from cytotoxic lymphocytes. This cleavage results in the removal of their C-terminal domain inhibitors, leading to constitutive kinase activity. Additionally, mechanical signals from ECM such as elevated matrix stiffness or integrin-mediated adhesion, can stimulate RhoA functioning, thereby connecting extracellular biophysical cues to intracellular contractile signaling through the Rho/ROCK pathway [97,102]. In BC, activation of ROCK promotes increased fibroblast infiltration into the TME, driven by paracrine signals released by malignant cells [107]. MDA-MB-231, a well-defined TNBC cell line, which is known for its invasive and metastatic properties and lacks E-cadherin, treated with ROCK inhibitor (Y-27632 and Fasudil, 30 μM), formed clusters that possessed a less aggressive phenotype. Inhibition of ROCK in MDA-MB-231 cells reduced the phosphorylation level of MLC as well as the expression of integrin β1, GLUT3, ROCK and RhoA proteins. As a result, a significant reduction in disorganized actin-stress fibers, and restored basal polarity were noticeable [108]. Another study also demonstrated that Fasudil markedly reduced MDA-MB 231 cell migration and led to partial actin filaments disorganization, resulting in a reduced in vitro invasiveness [109]. The effect of inhibiting the Rho/ROCK signaling pathway on metastatic traits in TNBC cells was further examined in relation to cellular mechanical properties using atomic force microscopy. Morphological and biomechanical changes were assessed by measuring Young’s modulus [110], a parameter increasingly recognized as a key biomarker capable of differentiating normal from malignant cells or tissues based on nanoscale stiffness variations [111]. Results indicate the MDA-MB-231 cell line has lower parameters compared to normal breast cells. Furthermore, it is the highest among other subtypes of BC cell lines [112]. The analysis of Young’s modulus parameter demonstrated that the inhibition of ROCK decreased the elasticity of MDA-MB-231 in both analyzed cellular components (cytoplasm and nucleus) equally. As a consequence, the enhancement of cellular rigidity induced by Y-27632 treatment could also prevent the relapse formation process in cells with a high invasive potential [110].

On the other hand, several studies show that inhibition of RhoA actually drives migration and invasion, eventually leading to successful metastasis. A study of Rho GTPase-activating protein 18, a member of the RhoGAP family, revealed that it promotes metastasis by downregulating RhoA signaling. This suggests that inhibiting RhoA has a metastatic effect [95]. In the study where the highly metastatic 4T1 orthotopic mouse model, well known for its close resemblance to human TNBC, was used to demonstrate that suppressing RhoA expression enhances BC metastasis to the lungs, mechanistically, the findings indicate that reduced RhoA levels lead to upregulation of the chemokine receptors CXCR4 and CCR5, thereby increasing the capacity of tumor cells to migrate toward the sentinel lymph node, gain access to the circulatory system, and subsequently disseminate to distant sites [113]. Another study on Raf-1 kinase inhibitor protein (RKIP), which was initially discovered as a physiological kinase inhibitor of the MAPK signaling pathway and was later shown to suppress cancer cell invasion and metastasis, also demonstrated a probable involvement of RhoA in RKIP-mediated suppression of BC cell invasion [114]. Those data suggest a dual role of the Rho/ROCK signaling pathway in cancer cells’ invasion. 

In the context of metastatic progression, Rho/ROCK signaling plays a critical role in regulating cytoskeletal dynamics, cell contractility, and mechanical properties of tumor cells, particularly during invasion, intravasation, and extravasation. Experimental studies have demonstrated that activation of this pathway is associated with increased actin stress fiber formation, enhanced focal adhesion assembly, and changes in cellular stiffness, all of which contribute to invasive behavior. Functionally, Rho/ROCK signaling promotes tumor cell migration and invasion, as evidenced by Transwell and wound-healing assays, and facilitates transendothelial migration and metastatic dissemination in in vivo models. These findings highlight the importance of Rho/ROCK signaling in linking biomechanical properties of tumor cells with metastatic competence, particularly in processes requiring active cell movement and tissue penetration [108,109,110,112].

Overexpression of several molecules (Table 1) has been linked to enhanced metastatic behavior in TNBC via Rho/ROCK pathway modulation, including nuclear factor erythroid 2-related factor 2 (NRF2) [115] and latent TGF-β-binding protein 1 (LTBP1) [116]. NRF2, a transcription factor aberrantly activated in various BC cells, promotes metastasis by activating RhoA, which functions as a downstream effector. Activation of NRF2 stimulates RhoA-dependent signaling, enhancing stress fiber assembly and focal adhesion formation, ultimately facilitating metastatic progression [115]. LTBP1, known for modulating the bioavailability of TGF-β, participates in regulating cell proliferation, migration, and apoptosis, indicating its relevance in tumorigenesis. Transwell assays demonstrated that silencing LTBP1 markedly reduced the migratory capacity of TNBC cells. Knockdown of LTBP1 also suppressed EMT, as shown by increased epithelial marker expression and decreased mesenchymal marker expression. Additionally, LTBP1 depletion significantly lowered RhoA activity and reduced phosphorylation of MLC2 at Ser19, indicating inhibition of the RhoA/ROCK pathway. These findings collectively suggest that RhoA/ROCK signaling acts downstream of LTBP1 in TNBC [116].

### 4.2. PI3K/Akt Signaling Pathway

The PI3K/Akt signaling cascade represents a central regulatory pathway across numerous cancer types [98]. It governs several core cancer hallmarks, including cell survival, metabolic reprogramming, and metastatic behavior [118]. Within the TME, PI3K/Akt also contributes to EMT, angiogenesis and to the recruitment of inflammatory mediators. Furthermore, it is also essential for the survival of circulating tumor cells (CSCs), which are important in metastasis formation [119]. Activation of this pathway can be triggered by multiple upstream factors, such as RTKs, Toll-like receptors (TLRs), and B-cell antigen receptors (BCRs). Its dysregulation is frequently driven by various genomic alterations, including mutations in PI3KCA, PTEN, Akt, TSC1, and mTOR. Mechanistically, PI3K catalyzes the phosphorylation of phosphatidylinositol-4,5-bisphosphate (PIP2) to produce phosphatidylinositol-3,4,5-trisphosphate (PIP3), which in turn recruits several oncogenic effectors, most notably the serine/threonine kinase Akt. When activated, Akt phosphorylates multiple downstream targets containing the consensus motif R-X-R-X-X-S/T. Among its major downstream mediators, mTOR serves the most prevalent downstream effector, integrating diverse proteins to drive tumor progression [120,121]. Almost 70% of BC cases demonstrate an abnormally altered PI3K/AKT/mTOR signaling pathway. Alterations or mutations in the PI3K/AKT/mTOR pathway occur in 25% of primary TNBC and possibly more frequently in metastatic TNBC [122]. A study provided in vivo evidence that PI3K/mTOR signaling plays a crucial role in metastatic progression in a bone metastasis model. The high-bone-relapse MDA-MB-231 subline 1833 exhibited activated PI3K/mTOR signaling, elevated levels of p27 phosphorylated at T157 and T198, and p27-dependent motility and invasion in vitro. Treatment with PF-04691502, a catalytic PI3K/mTOR inhibitor, reduced p27 phosphorylation and its cytoplasmic localization, thereby suppressing tumor cell invasiveness. Importantly, the inhibitor also markedly impaired the expansion of bone metastases in vivo. Conversely, expression of a p27CK-T157D/T198D mutant caused cell resistance to PF-04691502-mediated inhibition of migration and invasion, indicating that the drug’s effects are partially mediated through p27. Collectively, these findings identify PI3K/mTOR signaling as a central driver of metastatic behavior in TNBC [123,124]. There are also many other inhibitors associated with this pathway that can significantly treat TNBC and are clinically useful, which underlines the importance of this signaling pathway in tumor progression [122,125]. The PI3K/Akt/mTOR signaling pathway promotes EMT. Inhibiting mTOR with agents such as INK128, AZD8055, or rapamycin markedly reduces the abundance of Snail, a key EMT-inducing transcription factor in the TNBC cell line. Evidence indicates that mTOR regulates Snail through two distinct mechanisms: mTORC1 mainly increases its translation, whereas mTORC2 helps maintain Snail stability by slowing its degradation. Together, these observations establish a mechanistic link between mTORC2 activity and the upregulation of EMT and invasive cell behavior [126].

In the context of metastatic progression, PI3K/Akt signaling plays a critical role in regulating multiple stages of the metastatic cascade, particularly cell survival, metabolic adaptation, and resistance to apoptosis during circulation and colonization. Experimental studies have demonstrated that activation of this pathway is associated with downregulation of epithelial markers such as E-cadherin and upregulation of mesenchymal markers including N-cadherin and vimentin, indicating induction of epithelial–mesenchymal transition. Furthermore, PI3K/Akt signaling enhances tumor cell migration and invasion, as evidenced by Transwell and wound-healing assays, and contributes to increased metastatic potential in vivo, as demonstrated in xenograft and orthotopic breast cancer models. These findings collectively support a stage-specific role of PI3K/Akt signaling in metastatic progression, linking molecular activation of the pathway with functional and experimentally validated outcomes [123,124,126].

Among the molecules which activate PI3K/Akt (presented in Table 2) and predispose to metastasis, there are nuclear factor erythroid 2-related factor 3 (Nrf3) [127], actin-like protein 8 (ACTL8) [128], and receptor tyrosine kinase-like orphan receptor 2 (ROR2) [129]. All of them are overexpressed in TNBC and associated with poor prognosis. Detailed studies of the effects of Nrf3 and ROR2 have shown that overexpression of both proteins leads to upregulation of N-cadherin and vimentin, along with downregulation of E-cadherin. Additionally, Nrf3 upregulates MMP3 and 9, while ROR2 upregulates MMP2 and Snail. All these processes occur via the PI3K/Akt pathway. These molecules are key mediators of TNBC metastasis, with a specific impact on promoting EMT.

### 4.3. TGF-β Signaling Pathway

TGF-β is a signaling molecule belonging to the group of multifunctional cytokines with diverse functions. Typically, cytokines mediate cell communication and immune responses, but TGF-β has a much broader effect during both embryonic and adult life [132]. This includes differentiation, wound healing, proliferation, and regulation of cell- and tissue-specific motility. TGF-β signaling in breast cancer metastasis enhances local invasion and dissemination by inducing EMT and promoting a shift from collective to single-cell motility. Additionally, it is engaged in the extravasation process of CTCs [65]. Within the TGF-β family, there are ligands such as TGF-βI, which is considered the most abundant and ubiquitously expressed in humans, TGF-βII, and TGF-βIII [99]. It is synthesized as a dimer. This cytokine is composed of two basic elements: a large N-terminal pro-domain referred to as the latency-associated peptide (LAP), which prevents TGF-β activation, and a short mature peptide C-terminal domain [65]. In order to bind to the receptor, the latent TGF-β needs to be activated. This occurs when mature TGF-β gets cleaved and the LAP element is dissociated. There are several ways this can happen. In vitro conditions like heating of TGF-β with mild acid, lowering the pH to 4.5 or by oxidative modification, where there are reactive oxygen species (ROS), may lead to this. The in vivo cleavage includes the proteolytic cleavage of LAP via various ECM serine proteases, i.e., plasmin, leucine-rich repeat consisting of protein 33, MMPs, i.e., MMP9 and MMP14, cathepsin D and thrombospondin-1, which release the active TGF-β. TGF-β signaling can be transmitted through two main mechanisms. In the canonical pathway, active TGF-β ligands first bind to the type II TGF-β receptor (TβRII), which subsequently recruits and phosphorylates the type I receptor (TβRI). Ligand binding and receptor complex formation activate the kinase function of TβRII, enabling it to trans-phosphorylate specific serine/threonine residues within the GS domain and juxtamembrane region of TβRI. Once activated, TβRI initiates intracellular signaling primarily through members of the SMAD protein family [99,133]. The SMAD protein family is divided into three functional categories: (i) the R-SMADs (receptor-regulated SMADs), including SMAD1, SMAD2, SMAD3, SMAD5 and SMAD8 (also known as SMAD9), each mediating a particular signaling pathway; (ii) the Co-SMADs (co-mediator SMADs), including SMAD4 that mediates signaling by diverse TGF-β family members; and (iii) the inhibitory SMADs, which include SMAD6 and 7, which attenuate SMAD-dependent pathways by promoting receptor degradation or by competing with R-SMADs for binding to activated receptor complexes [134,135]. Upon activation, TGF-βRI phosphorylates R-SMADs, predominantly SMAD2 and SMAD3. The phosphorylated R-SMADs then associate with the Co-SMAD4 to form a complex that translocates into the nucleus, where it modulates the transcription of target genes. In addition to this canonical SMAD-dependent signaling, TGF-β can also initiate several non-canonical (SMAD-independent) pathways in specific cell types through TGF-β receptor activation. These alternative pathways include regulation of actin cytoskeleton dynamics and cell motility through Rho-like GTPases; modulation of cell migration and tight junction integrity via PAR6; control of cell proliferation, survival, and metastatic behavior through ERK/MAPK and PI3K/Akt signaling; promotion of cell migration through the Rho/ROCK axis; and regulation of immune evasion, cell survival, and inflammatory responses via the nuclear factor-κB (NF-κB) pathway [131,133].

The role of TGFβ in cancer progression is ambiguous. In the initial stages, it acts as a tumor suppressor. However, as the disease develops, it transforms into a tumor promoter, driving tumorigenesis that undergoes EMT and eventually leads to chemoresistant metastatic cancer [136,137]. This dual role is also observed in BC [138]. A study conducted in 2016, with 80 samples from TNBC patients, showed that intratumoral high expression of TGF-β1 was observed in TNBC (52.5%) compared with non-TNBC (27.5%), implying a probable role for TGFβ signaling in TNBC biology. Tests assessing the invasive and migratory abilities of TNBC cells showed that the addition of TGF-βI significantly increased these properties compared with the control group. Based on these results, it can be concluded that high expression levels of TGF-βI may serve an important role in promoting TNBC development. Moreover, this cytokine may contribute to the high malignancy and high rate of metastasis and recurrence of TNBC. Following treatment of MDA-MB-231 cells with TGF-βI, the protein expression levels of P38 and SMAD2 were increased, as well as their corresponding phosphorylated proteins, suggesting that the P38 pathway (belonging to SMAD-independent TGF-β signaling) and SMAD2 pathway may serve important roles in the effects of TGF-β1 on promoting cell invasion and migration [134,139]. Another study revealed that high serum levels of TGF-βI were significantly associated with a high incidence of metastasis, recurrence and a poor response to treatment (FEC100 treatment, which included Fluorouracil, Epirubicin and Cyclophosphamide). It was elucidated that although BC tumor tissue exhibits higher levels of expression of TGF-βI than the corresponding normal tissues, the association of TGF-βI with cancer is strongest in advanced disease stages. This pattern is consistent with the dual, stage-dependent nature of TGF-βI signaling in BC. At early stages of tumorigenesis, TGF-βI acts predominantly as a tumor suppressor by regulating the balance between cellular proliferation, differentiation, and programmed cell death. As carcinogenesis progresses and this regulatory balance deteriorates, two major shifts occur. First, there is a broad reduction in TGF-β receptor-mediated signaling, which diminishes the growth-inhibitory effects of TGF-βI. Second, tumors begin to overproduce active TGF-βI, driving a transcriptional program that promotes invasion, angiogenesis, and metastatic dissemination. This shift ultimately fosters the emergence of a mesenchymal, highly aggressive cellular phenotype. This model provides a mechanistic explanation for the observed link between TGF-βI overexpression and increased metastatic burden and recurrence. Furthermore, decreased expression of TGF-β receptors elevates TGF-βI concentrations within the tumor microenvironment, removing its early tumor-suppressive influence and facilitating invasive behavior in most breast cancer cases [140,141]. In the work of Fei Huang et al., HER2/EGFR signaling was shown to redirect TGF-β activity in BC, enabling AKT-dependent phosphorylation of Smad3 and thereby stimulating EMT and cell migration [142]. TGF-β also interacts with the Wnt pathway through β-catenin. Anders Sundqvist et al. demonstrated that TGF-β induces the expression of Wnt7a and Wnt7b via Smad2/3, which further amplifies TGF-β–driven EMT in mammary epithelial cells. Consistently, Wnt pathway components were highly represented among late-phase TGF-β-responsive genes [143]. Integrating microRNA sequencing of human TNBC tissues with matched normal samples and follow-up experiments in TNBC cell lines revealed that growth differentiation factor-10 (GDF10), a TGF-β superfamily member, is significantly reduced in TNBC. This loss of GDF10 was strongly associated with enhanced invasive behavior. Western blot analyses further showed that restoring GDF10 expression decreased levels of p-Smad2, N-cadherin, and vimentin, while increasing Smad7 and E-cadherin. Immunofluorescence studies demonstrated that GDF10 overexpression also reduced the nuclear localization of Smad4. Collectively, these findings indicate that GDF10 acts as a tumor-suppressive factor in mammary epithelial cells by inducing cell cycle arrest and blocking EMT [144]. The cellular morphology of cancerous breast cell lines undergoes a transformation from a oval-like shape to a thin, elongated spindle-like form, referred to as EMT, after treatment with TGF-β [144,145,146]. Expression of E-cadherin is reduced in breast cells after treatment with TGF-β. The level of vimentin has been shown to enhanced under the exposure to TGF-β [146,147]. Cells treated with mature TGF-βI demonstrated an increase in integrin αv and integrin α6 expressions [148]. The progression of metastasis can also occur by upregulating the expression of partitioning-defective protein 6 (Par6) through TGF-β-dependent signaling. This protein is responsible for cell polarity regulation and junction stability maintenance. In BC, Par6 promotes the loss of polarity and induces mesenchymal-like invasive mammary tumor cells. Notably, studies have shown that by blocking Par6 signaling, the EMT process can be arrested [149].

In the context of metastatic progression, TGF-β signaling plays a central and stage-specific role, particularly in the induction of epithelial–mesenchymal transition, as well as in the regulation of invasion, intravasation, and extravasation. Experimental studies have demonstrated that activation of TGF-β signaling leads to downregulation of epithelial markers such as E-cadherin and upregulation of mesenchymal markers including vimentin and N-cadherin, accompanied by morphological changes characteristic of a mesenchymal phenotype. Functionally, TGF-β enhances tumor cell migration and invasion, as shown in Transwell and wound-healing assays, and promotes metastatic dissemination in vivo, as demonstrated in orthotopic and xenograft breast cancer models. These findings highlight the role of TGF-β as a key regulator of metastatic plasticity, linking pathway activation to experimentally validated changes in tumor cell behavior across multiple stages of the metastatic cascade [134,139,140,144].

Studies indicate that a diverse set of upstream regulators can influence metastatic progression in TNBC through TGF-β-dependent signaling. Several factors have been identified as promoters of metastatic relapse (Table 3.), including YTH domain-containing protein 1 (YTHDC1) [150], intercellular adhesion molecule 1 (ICAM1) [151], transmembrane protein 158 (TMEM158) [152], mitochondrial phosphoenolpyruvate carboxykinase (PEPCK-M) [153], karyopherin-α3 (KPNA3) [154], and NAD(P)H steroid dehydrogenase-like protein (NSDHL) [155]. Metastatic dissemination driven by these molecules is commonly associated with increased expression of N-cadherin, vimentin, and EMT-related transcription factors such as Snail, ZEB, and Twist1, together with reduced E-cadherin levels and loss of epithelial polarity, ultimately promoting a mesenchymal phenotype. In contrast, Sirtuin 7 (SIRT7) [156], miR-190 [157], and the 5-hydroxytryptamine receptor 1A (HTR1A) [158] function as metastasis suppressors. Their upregulation inhibits metastatic progression primarily by attenuating EMT, thereby exerting protective effects against TNBC dissemination. A more detailed description is provided in Table 3.

### 4.4. Wnt/β-Catenin Signaling Pathway

The Wnt/β-catenin signaling pathway plays a crucial role both during embryonic development and in maintaining homeostasis in the adult organism [164]. Its primary functions include regulating various cell fate determinations, including proliferation, migration, polarity, and apoptosis [100]. In the process of metastasis in breast cancer, it is of particular importance at the stage of EMT, promotion of stem-like features and colony formation [56]. Signal transmission occurs through one of three pathways: the canonical Wnt/β-catenin signaling pathway, the “non-canonical” Wnt/Ca2+ signaling pathway, and the Wnt/planar cell (PCP) signaling pathway [165]. In the canonical pathway, β-catenin is transported to the cell nucleus, where it activates target genes via β-catenin-T-cell factor/lymphoid enhancer-binding factor (TCF/LEF) transcription factors. These are primarily involved in controlling proliferation, in contrast to noncanonical Wnt pathways, which perform functions such as cell polarity and migration regulation independently of TCF/LEF. Furthermore, in some mammalian tissues, Wnt signaling is responsible for self-renewal. It is worth noting that these two main pathways form a network of mutual regulation. In the absence of Wnt ligands, the transmembrane receptors FZD and LRP5/6 remain separated on the cell surface. Meanwhile, in the cytoplasm, β-catenin is continuously targeted for degradation by a “destruction complex” composed of adenomatous polyposis coli (APC), AXIN, casein kinase 1 (CK1), and glycogen synthase kinase 3 (GSK3). CK1 and GSK3 sequentially phosphorylate β-catenin, marking it for proteasomal breakdown. When Wnt ligands bind simultaneously to FZD and LRP5/6, this interaction recruits the destruction complex to the plasma membrane, disrupting its ability to degrade β-catenin. As a result, β-catenin accumulates in the cytoplasm and subsequently moves into the nucleus, where it interacts with TCF/LEF transcription factors to activate Wnt target genes. The movement of β-catenin from the cytoplasm to the nucleus is a crucial event indicating activation of the Wnt/β-catenin signaling pathway [166,167,168]. mRNA profiling of formalin-fixed, paraffin-embedded TNBC samples revealed that activation of the Wnt/β-catenin pathway is strongly linked to higher tumor grade, unfavorable clinical outcomes, and metastatic progression. Within the TNBC subtype, elevated Wnt signaling correlates specifically with an increased likelihood of lung metastasis. Experimental inhibition of the Wnt/β-catenin cascade, using pharmacologic antagonists such as WntC59 or sulindac sulfide, or through β-catenin–targeted siRNA, demonstrated that disrupting this pathway directly suppresses metastasis-related phenotypes. These include reduced fibronectin-guided migration, impaired F-actin cytoskeletal remodeling, and diminished invasive capacity in TNBC cells [168]. Further studies also showed that TNBC cells acquire migratory, invasive and clonogenic properties via upregulation of Wnt signaling. Thus, its upregulation observed in tumors of TNBC patients is functionally associated with metastasis-associated phenotypes [169]. CSCs are a group of cells that express EMT markers, which facilitate tumor cells to acquire migration abilities to invade other tissues or organs [170]. More specifically, several studies propose that metastasis-initiating cells may arise within small subpopulations of CSCs [171]. For instance, CD44+/CD24low BC cells, which possess stem-like features, have been shown in xenograft models to exhibit elevated tumor-initiating capacity and increased metastatic potential [172,173]. Growing evidence also highlights a central role for Wnt/β-catenin signaling in regulating CSC biology [174]. Mammary stem cells with high Wnt/β-catenin activity display markedly greater tumorigenicity than those with low pathway activity. Consistent with this, shRNA-mediated silencing of Wnt1 in 4T1 cells leads to reduced expression of stem cell markers such as ALDH1, Sca-1, and CD44+/CD24-, decreased formation of breast cancer stem cell spheres, decreased proliferation both in vitro and in vivo and impaired migratory capacity in vitro and in vivo. Collectively, these findings demonstrate that Wnt/β-catenin signaling drives lung tumorigenesis by enhancing tumor growth, reshaping CSC phenotypes, and promoting cancer cell invasion and dissemination. Thus, Wnt/β-catenin signaling is a key regulator of CSC self-renewal and motility, ultimately benefiting metastatic spread in BC [175].

In the context of metastatic progression, Wnt/β-catenin signaling plays a stage-specific role, particularly in the regulation of epithelial–mesenchymal transition, cancer stem cell maintenance, and metastatic colonization. Experimental studies have demonstrated that activation of this pathway is associated with downregulation of epithelial markers such as E-cadherin and increased expression of mesenchymal markers including N-cadherin and vimentin, consistent with EMT induction. Functionally, Wnt/β-catenin signaling enhances tumor cell migration, invasion, and clonogenic potential, as shown in Transwell and sphere-formation assays, and promotes metastatic tumor growth in vivo in xenograft models. These observations highlight the critical role of Wnt/β-catenin signaling in linking stem-like properties with metastatic competence, particularly during colonization and outgrowth at secondary sites [168,169,175].

The Wnt signaling cascade is tightly controlled by numerous upstream regulatory molecules, listed in Table 4, that either potentiate or suppress its activity, thereby influencing metastatic progression in TNBC in a variety of ways. Consistent with previously described modulators of signaling pathways, activation of the Wnt pathway promotes relapse and metastatic dissemination primarily through the upregulation of EMT-associated transcription factors, including Snail, ZEB, and Twist1. This transcriptional shift drives increased expression of mesenchymal markers including N-cadherin, vimentin, and fibronectin, accompanied by a reduction in E-cadherin levels. Conversely, inhibition of Wnt signaling by specific molecular regulators reverses these effects, supporting the maintenance of an epithelial phenotype and suppressing EMT-dependent metastatic traits.

### 4.5. NF-κB Signaling Pathway

NF-κB is group of transcription factors that regulate the expression of genes involved in numerous biological functions, such as inflammatory and immune responses, as well as cellular proliferation and survival [204]. However, NF-κB signaling also contributes to multiple stages of TNBC metastasis. It induces EMT, drives angiogenesis, promotes tumor cell invasion and induces [205] an inflammatory state in PMN to promote metastatic colonization [85]. This family comprises five proteins, RelA/p65, c-Rel, RelB, NF-κB1 (p50), and NF-κB2 (p52), which can assemble into various homo- or heterodimeric complexes to mediate context-dependent signal transduction [206]. Due to the presence of a conserved domain known as the Rel-homology domain, all proteins belonging to this family have the ability to form dimers, transport to the cell nucleus, bind DNA and interact with the IkB protein inhibitors [207]. After binding to IκBs, NF-κB remains sequestered in the cytoplasm as an inactive complex. Activation occurs through canonical or noncanonical pathways triggered by immune stimuli. In the canonical pathway, proinflammatory cytokines such as TNF-α and IL-1β, bacterial lipopolysaccharide, and antigens activate receptor-proximal signaling that stimulates the IKK complex (IKKα, IKKβ, and NEMO/IKKγ). Activated IKK phosphorylates IκB proteins (mainly IκBα), leading to their ubiquitin-dependent proteasomal degradation. This releases NF-κB dimers (typically p50/RelA), which translocate to the nucleus and activate target gene transcription. The noncanonical pathway is mainly triggered by TNF receptor (TNFR) superfamily members. Ligand binding disrupts an E3 ubiquitin ligase complex (TRAF2, TRAF3, and c-IAP1/2) that normally targets NF-κB-inducing kinase (NIK) for degradation. When this complex is disrupted, NIK stabilizes and accumulates, activating IKKα, which phosphorylates p100 (NF-κB2 precursor). This phosphorylation causes ubiquitin-dependent processing of p100 to p52, allowing p52/RelB dimers to enter the nucleus. The noncanonical pathway regulates specialized immune functions, including lymphoid tissue development, B-cell maintenance, and T-cell activity. Both pathways also contribute to metastasis, promoting EMT via TWIST1 and SNAIL, and stimulating angiogenesis through increased VEGF and IL-8 expression [101,208].

In their studies on BC, Prajoko and Aryandono demonstrated that NF-κB expression significantly increases the risk of metastasis in advanced disease. The results showed that this transcription factor upregulates the expression of MMPs, urokinase-type plasminogen activator, and cytokines, which are molecules responsible for the highly metastatic and aggressive properties of BC. Furthermore, an increase in the expression of the G-protein-coupled chemokine receptor was detected, which strongly increased the motility of BC cells [209]. Other studies have demonstrated that TNF-α/NF-κB/TWIST1 signaling axis promotes EMT, suggesting that therapeutic targeting of this axis may impede metastasis [101,210]. TGF-β1 is a central inducer of EMT and is known to suppress E-cadherin expression, elevate mesenchymal markers, and promote a mesenchymal-like morphology characterized by the breakdown of intercellular junctions. Lee et al. demonstrated that exposure of TNBC cells to TGF-β1 leads to robust activation of IκBα and NF-κB, accompanied by marked upregulation of the EMT-associated transcription factors Snail and Slug, as well as a pronounced reduction in E-cadherin expression. Collectively, these observations suggest that the loss of epithelial junctional integrity, primarily driven by E-cadherin downregulation, is functionally connected to enhanced NF-κB signaling during EMT, either through increased nuclear translocation of NF-κB or through elevated expression and/or activity of NF-κB components [211]. After using (S)-b-salicyloylamino-a-exo-methylene-ƴ-butyrolactone, an inhibitor for the NF kB pathway, a strong reduction in invasive capacity mediated by MMP2 and vimentin decrease was demonstrated by cell invasion and wound healing assays [212].

In the context of metastatic progression, NF-κB signaling plays a stage-specific role, particularly in the regulation of inflammation, epithelial–mesenchymal transition, and immune evasion. Experimental studies have demonstrated that activation of NF-κB is associated with downregulation of epithelial markers such as E-cadherin and upregulation of mesenchymal markers including vimentin and N-cadherin, reflecting induction of epithelial–mesenchymal transition. Functionally, NF-κB signaling enhances tumor cell migration and invasion, as evidenced by Transwell and wound-healing assays, and promotes metastatic dissemination and survival through the regulation of inflammatory cytokines and immune-modulatory factors. These findings highlight the dual role of NF-κB signaling in linking inflammatory signaling with metastatic progression, particularly by facilitating immune escape and supporting colonization at secondary sites [109,110,111,112,113,114,115,116,117,118,119,120,121,122,123,124,125,126,127,128,129,130,131,132,133,134,135,136,137,138,139,140,141,142,143,144,145,146,147,148,149,150,151,152,153,154,155,156,157,158,159,160,161,162,163,164,165,166,167,168,169,170,171,172,173,174,175,176,177,178,179,180,181,182,183,184,185,186,187,188,189,190,191,192,193,194,195,196,197,198,199,200,201,202,203,204,205,206,207,208,209,210,211,212].

Evidence shows that a broad range of upstream regulators can modulate metastatic progression in TNBC via NF-κB-dependent signaling. Several factors have been presented and described in Table 5.

## 5. Extracellular Matrix in TNBC

The ECM is an essential element of every tissue that built up the body, consisting of an intricate network of molecules that determine both structural integrity and the biochemical and biomechanical properties of the tissue [220]. In terms of composition, the ECM is primarily formed by structural proteins (e.g., collagen and elastin), glycosaminoglycans, proteoglycans, and adhesive molecules such as fibronectin and laminin. Structurally, it encompasses the interstitial connective matrix, which provides mechanical support, and the basement membrane, which separates epithelial layers from the underlying stroma and maintains epithelial and endothelial cell organization. Functionally, the ECM governs key cellular processes including adhesion, migration, and differentiation. Moreover, it modulates multiple signaling pathways that regulate cell survival, proliferation, and tissue morphogenesis [221,222,223].

The structural protein, that is most abundant in the ECM is collagen [224]. It constitutes approximately 25–30% of human protein mass [225]. During tumor development, collagen fibers that are initially thin, loose, disorganized, and wavy gradually become more linear, crosslinked, and thickened. They reorient perpendicularly to the tumor edge and generate tension on adjacent epithelial cells. These structural alterations increase ECM rigidity and create aligned tracks that promote cancer cell migration toward blood vessels, thereby enhancing metastatic potential [226]. However, other reports indicate that it may constitute a physical barrier against invasion thus the role of collagen in metastasis is not clear [227]. Elevated levels of fibrillar collagen stimulate the release of ECM-degrading enzymes, thereby creating channels that facilitate cancer cell movement. High mammographic density (MD), defined by abundant fibrillar collagen, reduced adipose content, and an expanded stromal compartment, is linked to a four- to six-fold increase in BC risk and to more aggressive disease features, including larger primary tumors, greater lymph node involvement, and high-grade, high-stage, rapidly proliferating, and hormone-receptor-negative cancers. In addition to hindering tumor detection, elevated MD may actively promote tumor cell proliferation and expansion. Dense breast tissue is associated with greater collagen deposition, alignment, linearity, stiffness, organization, and fiber thickness, and these collagen characteristics may serve as biomarkers predictive of BC risk and tumor aggressiveness [228]. Among the different types of collagen, collagen type IV alpha 2 (COL4A2) is the major structural component of basement membranes. COL4A2 is significantly upregulated in TNBC, suggesting that it may play an important role in the pathogenesis of TNBC. When the expression of this protein is silenced in MDA-MB-231 and MDA-MB-468 cells after siRNA lentivirus transfection inhibition in cell migration is revealed, indicating the migration of TNBC cells is upregulated by Col4A2 expression [229]. Another type of collagen: collagen type VI alpha 3 chain, widely present in most connective tissues, is considered a predictive marker of poor prognosis in TNBC. Analysis of samples from TNBC patients showed that it is among 6 EMT genes to predict metastasis in this type of cancer [230]. Collagen type X alpha 1 (COL10A1) is markedly upregulated in multiple solid tumors, including TNBC. Functional assays demonstrate that increased COL10A1 expression enhances migratory capacity in the TNBC cell lines MDA-MB-231 and BT-549, whereas COL10A1 silencing suppresses this behavior. Elevated COL10A1 levels induce a shift toward a mesenchymal phenotype, quantified by increased N-cadherin and vimentin and reduced E-cadherin expression. Moreover, COL10A1 overexpression is associated with higher levels of β-catenin and phosphorylated GSK3β (Ser9), whereas decreasing COL10A1 reverses these molecular changes. Collectively, these alterations suggest that COL10A1 contributes to EMT in TNBC, potentially through activation of the Wnt/β-catenin signaling pathway [231]. Last but not least, collagen XIII protein (mostly expressed on the cellular membrane) is found to be expressed at higher levels in TNBC cell lines including MDA-MB-231, Hs578T, BT549, and T4–2 than in luminal-type BC cell lines and non-malignant mammary epithelial cell lines. Transwell experiments revealed that silencing collagen XIII significantly reduced invasion of MDA-MB-231 cells, while single-cell tracking analysis demonstrated that knockout of this protein reduced the velocity and the distance of cell migration. These results show that elevated expression of collagen XIII in MDA-MB 231 cells promotes malignant phenotypes in 3D culture by enhancing cancer cell migration and invasion. Further analysis revealed that collagen XIII enhances cell invasion and mammosphere formation at least partially through the β1 integrin pathway [232].

BC also shows increased levels of other ECM components, namely, FN and hyaluronic acid (HA). A 3D tumor model of TNBC was developed to study the effects of FN and HA, alone or in combination, on TNBC cell invasion of collagen-based hydrogels. Results showed that FN and HA significantly increase collagen invasion of TNBC cells and oncogenic signaling but not in combination, potentially due to differences in the microstructure of the hydrogels. Molecular analyses revealed that FN markedly enhances activation of the RhoA/ROCK1 signaling cascade, which regulates adhesion complex formation and stress fiber assembly to promote cell motility. FN also robustly increased MAPK/ERK pathway activity in SUM159 cells, an established driver of motility in TNBC, whereas this effect was not observed in MDA-MB-231 cells, likely due to their already elevated baseline ERK signaling. In contrast, HA did not alter ERK activity but instead induced the upregulation of CD44 receptors (a CSC marker) on TNBC cells [233].

The ability of cancer cells to invade surrounding tissue depends also on their capacity to break down the adjacent ECM, a process that not only supports tumor expansion but also compromises the integrity of normal structures [234]. Importantly, ECM destruction is accompanied by the formation of a changed, tumor-specific matrix that is typically more compact and mechanically rigid. These physical characteristics, such as increased stiffness, altered mechanical stress, and changes in interstitial fluid pressure, are known to regulate numerous cellular behaviors. Consequently, modifications in the ECM profoundly influence the metastatic potential of cancer cells [235]. Research has increasingly shown that the composition and orientation of ECM fibers, the degree of cross-linking within the matrix, and the presence of specific enzymes such as MMPs and tissue inhibitors of metalloproteinases (TIMPs) play crucial roles in these processes. MMPs belong to zinc-dependent enzymes that support the dynamic reorganization of tissues by participating in the regular turnover of matrix components under normal physiological conditions. They also participate in many important cellular processes including migration, differentiation, proliferation, angiogenesis, and apoptosis, by processing ECM components and shedding bioactive ECM fragments. Due to the wide range of members of this enzyme family, it can be classified into substrate specificity and domain structure. Collagenases (e.g., MMP1, MMP8, and MMP13) cleave fibrillar collagens which are essential for skeletal and connective tissue integrity. Gelatinases (MMP2 and MMP9) degrade denatured collagens and type IV collagen, facilitating angiogenesis and metastasis. Stromelysins (MMP3 and MMP10) target a broader range of ECM proteins, including proteoglycans, laminin, and fibronectin. Membrane-type MMPs such as MMP14 and MMP15, anchored to the cell membrane, are pivotal for pericellular proteolysis, supporting cellular invasion and migration [222,236]. MMPs are secreted by a variety of different cells and tissues, including connective tissue, proinflammatory, and uteroplacental cells, including fibroblasts, osteoblasts, endothelial cells, vascular smooth muscle, macrophages, neutrophils, lymphocytes, and cytotrophoblasts [237]. While MMPs were once known as drivers of cancer progression due to their ability to proteolytically degrade the ECM, subsequent studies have revealed a far more complex role. Several MMPs, notably MMP3 and MMP8, can act protectively, and the biological outcome of MMP activity is highly context specific. The same enzyme may either support or suppress tumor development depending on the cellular source and the particular phase of cancer in which it is expressed [238]. In TNBC, MMP1 [239], 3 [240], 7 [241], 9 [242], 12 [243], and 13 [244] are all very highly expressed, while MMP2 [245] and 27 are downregulated [246]. MMP1 expression is significantly higher in TNBC than in other BC subtypes. Moreover, comparing the invasiveness of shRNA-MMP1-transfected TNBC cells with shRNA-control-transfected TNBC cells revealed a significant reduction in the invasion. MMP1 can promote cancer metastasis through degradation of the extracellular matrix, angiogenesis, osteoclast activation and tumor cell invasion, facilitating angiogenesis and increasing ECM degradation, which are important processes for the invasive and migratory phenotype of metastatic breast cancer [239]. Similar properties of MMP1 were demonstrated using exosomes isolated from supernatant of MDA-MB-231, where mass spectrometry and Western blot revealed its presence [247]. Overexpression of MMP9 has been linked to poor overall survival in patients with TNBC and lymph node metastasis [248]. Elevated MMP2 expression in tumor tissue is associated with an increased risk of bone metastasis in BC patients, and its silencing significantly reduces the invasive and migratory potential of TNBC cells [249,250]. Pharmacological suppression of MMP2/9, such as with brucine, has been shown to impair the formation of vasculature-mimicking structures in TNBC, indicating that targeting these enzymes could be an effective approach to inhibit vascular mimicry [251]. Nevertheless, the precise role of MMP9 in BC progression remains ambiguous. While some studies have reported its pro-tumorigenic or pro-metastatic activity in vivo, these effects vary depending on the specific mouse model used [238]. In TNBC specifically, MMP9 expression generally appears to promote metastasis [252,253]. In the mammary gland, MMP7 is normally present in ductal and lobular epithelial cells. Elevated MMP7 levels are significantly associated with patient age, tumor size, TN status, and recurrence. Notably, MMP7 expression is higher in primary tumors of patients whose cancer metastasized to the brain and/or lungs compared to those who developed bone metastases [254]. This metalloproteinase is regulated, among others, by FOXC1, a forkhead box transcription factor, which was identified as a functionally important biomarker of BC aggressiveness. Expression of FOXC1 and MMP7 is significantly correlated in BC samples and cell lines at both the mRNA and protein levels. Transient expression of FOXC1 in nontransformed mammary epithelial cell lines resulted in significantly increased MMP7 expression and an MMP7-dependent increase in invasiveness. Silencing endogenous FOXC1 reduces MMP7 expression without decreased expression of other matrix metalloproteinases [255]. Overexpression of MMP10 has also been associated with BC pathogenesis. In TNBC cells, chrysin treatment leads to a marked downregulation of MMP10, while levels of other MMPs and TIMPs remain largely unchanged. Notably, MMP10 exhibits relatively broad substrate specificity compared to other MMPs and is capable of degrading a wide range of both matrix and non-matrix components, which contribute to TNBC metastasis properties [256].

In addition to MMPs, the second group of enzymes that significantly influence TNBC metastasis formation by ECM modulation are cathepsins [257]. Cathepsins are protease enzymes, which are classified into multiple families; for example, based on the catalysis mechanism there are serine, cysteine, and aspartyl proteases [258]. These enzymes are active in the low pH environment of lysosomes. There are 16 members of the cathepsin family in humans [259]. By degrading the ECM, cathepsins enable tumor cells to escape from the primary tumor and invade into the vasculature, which enables the formation of distant metastases [260]. The aspartyl protease cathepsin D, recognized as a marker of poor prognosis in TNBC, is overexpressed and excessively secreted within the TME, where it exerts pro-tumorigenic effects [261]. Elevated cathepsin D levels are shown to enhance BC cell migration, invasion, and metastasis by upregulating ICAM-1 both in vitro and in vivo [262]. Cathepsin B (CTSB) serves as a prognostic indicator in various cancers, including BC, with elevated expression in primary tumors correlating with poorer outcomes. RNA interference-mediated suppression of CTSB in tumor cells leads to reduced collagen I degradation in vitro and decreased bone metastasis in vivo. Likewise, intraperitoneal treatment with the selective CTSB inhibitor CA-074 effectively reduce metastatic spread in tumor-bearing mice [263]. However, a recent study indicated that CTSB may exert distinct effects on metastasis depending on the TNBC cell type. Using a 3D single-cell invasion assay, in which cells were embedded in gels containing collagen I alone or combined with FN or collagen IV, CTSB knockdown in MDA-MB-231 cells led to increased invasion speed and persistence in FN and Collagen IV, suggesting that CTSB acts as a suppressor of invasion in these cells. In contrast, CTSB depletion in MDA-MB-468 cells produced different outcomes, markedly reducing tumor cell invasion speed in Collagen I, FN, and Collagen IV, while leaving persistence unaffected [264]. Cathepsin S (CTSS) differs from other enzymes in this family. Unlike most cathepsins, under physiological conditions, CTSS expression is restricted to specific cells and tissues and its activity is maintained at neutral and moderately alkaline pH. Furthermore, the immature CTSS is uniquely known (among human cathepsins) to be autocatalytically processed at neutral pH, when in the presence of glycosaminoglycans [265,266]. CTSS is mainly found inside the lysosomal/endosomal compartments of antigen-presenting cells, such as B cells, macrophages, dendritic cells, but is also produced by epithelial cells, smooth muscle cells, endothelial cells, and neutrophils [267]. In the MDA-MB-231 xenograft model, elevated CTSS expression specifically promoted metastasis to the brain. CTSS was secreted by both tumor cells and TAMs within the TME, with expression levels varying according to tumor stage and cell type. During the early stages of metastasis, including seeding and outgrowth, tumor cells exhibited high CTSS expression, whereas TAMs showed lower levels. This pattern reversed during the later colonization phase, with TAMs expressing increased CTSS and tumor cells contributing lower amounts. In this model, CTSS-mediated cleavage of junctional adhesion molecule B facilitated transmigration across the blood–brain barrier, a critical step in metastasis. Pharmacological inhibition of CTSS reduced brain metastasis when administered before tumor seeding and maintained throughout tumor progression, but did not affect tumor burden once metastatic lesions were already established [268]. Further investigation of CTSS in TNBC found that invasion and metastasis of TNBC cells were decreased via downregulation or inhibition of CTSS and MMP9. Inhibition of tyrosine kinase Src, which controls expression of CTSS and MMP9 as an activator for both PI3K/Akt and Ras/Raf/ERK pathways, resulted in a reduction of growth, invasion and metastasis of TNBC cells [269].

The next family of proteins that is involved in modulating the ECM in TNBC is the lysyl oxidase (LOX) family. It is responsible for the conversion of lysine residues in collagen and elastin precursors into highly reactive aldehydes, which results in cross-linking and stabilization of ECM proteins, specifically the type I collagen and elastin, and regulates cell adhesion, motility and invasion [270]. In preclinical BC models utilizing TNBC cell lines, LOX secretion from tumor cells was demonstrated to play a pivotal role in pre-metastatic niche formation. LOX, produced by hypoxic primary tumor cells, accumulates alongside FN at future metastatic sites, where it crosslinks collagen IV within the basement membrane and promotes adhesion of CD11b+ cells. These adherent CD11b+ cells secrete MMP2, which degrades collagen IV, facilitating further invasion of CD11b+ cells into lung tissue and releasing collagen IV-derived chemoattractant peptides. These peptides enhance the recruitment of additional CD11b+ cells, establishing a positive feedback loop that drives increased accumulation of bone marrow-derived cells, ECM remodeling, and the establishment of the PMN [271]. Further studies also show that lysyl oxidase-like 2 expression is correlated with increased metastasis and poor survival in TNBC patients [272]. In a mouse xenograft model, developed using a TNBC cell line, knockdown of lysyl oxidase-like 4 (LOXL4) resulted in enhanced primary tumor growth and increased lung colonization. This knockdown was also associated with thickened collagen bundles within the tumors. Analysis of the BreastMark dataset revealed that low LOXL4 expression correlated with poorer overall survival in BC patients, with the strongest association observed in TNBC cases. These findings suggest that reduced LOXL4 expression drives ECM remodeling by promoting collagen synthesis, deposition, and structural reorganization, thereby facilitating tumor progression and metastasis and contributing to adverse clinical outcomes in TNBC [273].

## 6. Conclusions

TNBC remains one of the most aggressive and lethal breast cancer subtypes, largely due to its strong propensity for metastasis and the lack of effective targeted therapies. Growing evidence indicates that metastatic progression in TNBC is driven by the coordinated dysregulation of several key signaling pathways, including Rho/ROCK, PI3K/Akt, TGF-β, Wnt/β-catenin, and NF-κB. These pathways collectively regulate essential processes associated with tumor dissemination, such as cell motility, invasiveness, survival, and the acquisition of stem-like phenotypes. In many cases, their pro-metastatic effects are mediated through activation of EMT, which enables tumor cells to acquire increased plasticity and migratory capacity, thereby facilitating metastatic spread. These pathways also play a central role in tumor progression and metastatic spread across multiple other cancers, including lung, colorectal, pancreatic, prostate, and hepatocellular carcinomas. Their broad, conserved roles make them key targets for anti-metastatic therapies across diverse malignancies.

Metastatic competence in TNBC is further enhanced by extensive remodeling of the ECM, driven by enzymes such as MMPs, cathepsins, and members of the LOX family. These proteolytic and crosslinking enzymes degrade structural barriers, increase tissue stiffness, and promote the formation of a tumor-supportive microenvironment that facilitates local invasion and colonization of distant organs.

Overall, the interplay between pro-metastatic signaling pathways, EMT activation, and ECM remodeling forms a complex and tightly interconnected network that underlies the aggressive phenotype of TNBC. A deeper understanding of these mechanisms may reveal novel therapeutic vulnerabilities and support the development of more effective strategies to prevent or limit metastatic progression in TNBC.

Emerging technologies, including single-cell omics and spatial transcriptomics, are expected to provide deeper insight into the cellular heterogeneity and signaling dynamics underlying TNBC metastasis. In parallel, physiologically relevant experimental models such as patient-derived organoids and microfluidic tumor-on-chip platforms may accelerate the identification of novel therapeutic targets and support the development of more effective precision medicine strategies.

## Figures and Tables

**Table 1:** Molecules that regulate Rho/ROCK signaling pathway in TNBC metastasis process.

Signaling Pathway	Upstream Regulator (Protein, RNA, Gene)	General Function	Expression in TNBC	Influence on Metastasis Formation in TNBC	References
Rho/ ROCK	NRF2 (nuclear factor erythroid 2-related factor)	Transcription factor: controls the expression of genes related to drug detoxification and the oxidative stress response, which in turn controls cellular defense against toxic and oxidative assaults	Overexpressed	↑ formation of stress fiber and focal adhesion	[115,117]
	LTBP1 (latent TGF-β binding protein 1)	Regulate the bioavailability of TGF-β; participate in cell proliferation, migration, and apoptosis	Overexpressed	↑ mesenchymal markers; ↓ epithelial markers; ↑EMT; ↑MCL2 phosporylation	[116]

Legend: ↑ activation or increase; ↓ inhibition or decrease.

**Table 2:** Molecules that regulate PI3K/Akt signaling pathway in TNBC metastasis process.

Signaling Pathway	Upstream Regulator (Protein, RNA, Gene)	General Function	Expression in TNBC	Influence on Metastasis Formation in TNBC	References
PI3K/Akt	ACTL8 (actin-like protein 8)	Cell proliferation, differentiation and motility	Overexpressed	↑ invasion and metastasis	[128]
	NRF3 (nuclear factor erythroid 2-related factor 3)	Transcription factor; involved in many bioregulation processes, like cell differentiation, inflammatory responses, oxidative stress, and lipid metabolism	Overexpressed	↑ N-cadherin, vimentin, MMP3, and MMP9 expression; ↓ E-cadherin expression	[127,130]
	ROR2 (receptor tyrosine kinase-like orphan receptor 2)	Regulating skeletal and neuronal development, cell polarity and migration	Overexpressed	↑ p-PI3K, p-AKT and p-mTOR; ↑ N-cadherin, vimentin, MMP-2 and Snail; ↓ E-cadherin	[129,131]

Legend: ↑ activation or increase; ↓ inhibition or decrease.

**Table 3:** Molecules that regulate TGF-β signaling pathway in TNBC metastasis process.

Signaling Pathway	Upstream Regulator (Protein, RNA, Gene)	General Function	Expression in TNBC	Influence on Metastasis Formation in TNBC	References
TGF-β	HTR1A (5-hydroxy-tryptamine receptor 1A)	GPCRs; most widely expressed and abundant subtypes of serotonin receptors; release of serotonin by acting both as a presynaptic autoreceptor in the serotonergic neurons of the dorsal and medial raphe nuclei and as a postsynaptic heteroreceptor in nonserotonergic neurons	Downregulated	↓ migration and invasion; ↓ expression of TβRII and p-Smad3	[158,159]
	KDM6B (lysine-specific demethylase 6B)	Development, differentiation, cell senescence and inflammation	Downregulated	↑ E-cadherin; ↓ fibronectin and vimentin; ↓ β-catenin; ↓ MMP-2, MMP-7 and MMP-9	[160]
	SIRT7 (Sirtuin 7)	rRNA transcription and modification, cellular metabolism, cellular stress, and DNA damage repair	Downregulated	↓ metastasis; ↓ cell migration; ↓ mesenchymal markers	[156,161]
	miR-190	Located at an intron region of the TLN2 gene	Downregulated	↓ SMAD2; ↑ E-cadherin expression; ↓vimentin and N-cadherin expression	[157]
	TMEM158 (transmembrane protein 158)	Oncogene	Overexpressed	Altered cell morphology (longer, fibroblast-like) ↑ N-cadherin and vimentin; ↑ transcription factors, such as ZEB1, SNAIL, Twist1; ↓ E-cadherin	[152]
	NSDHL (NAD(P)H steroid dehydrogenase-like protein)	Cholesterol biosynthesis	Overexpressed	↑ cell migration and invasion; ↑ TGFβR2 and p-SMAD3	[155]
	PEPCK-M (mitochondrial phosphoenolpy-ruvate carboxykinase)	Mitochondria-derived gluconeogenesis	Overexpressed	↑ SMAD3; ↑ N-cadherin and vimentin; ↑ transcription factors, such as ZEB1, SNAIL, Twist1; ↓ E-cadherin; ↑ EMT	[153,162]
	YTHDC1 (YTH domain-containing protein 1)	Nuclear m6A reader; identify many targets and mediate various RNA fates, including RNA degradation, nuclear export, alternative splicing, and RNA stabilization	Overexpressed	↑ mesenchymal marker IL11; ↑ mesenchymal morphology; ↑ SNAI1, FN; ↓ epithelial marker; ↑ EMT	[150,163]

Legend: ↑ activation or increase; ↓ inhibition or decrease.

**Table 4:** Molecules that regulate Wnt/β-catenin signaling pathway in TNBC metastasis process.

Signaling Pathway	Upstream Regulator (Protein, RNA, Gene)	General Function	Expression in TNBC	Influence on Metastasis Formation in TNBC	References
Wnt/β-catenin	CDK3 (cyclin-dependent kinase 3)	Involved in G0/G1 and G1/S cell cycle transitions	Downregulated	↓ invasion and metastasis	[176]
	RNF43 (ring finger protein 43)	E3 ubiquitin ligase	Downregulated	↓ EMT; ↓ c-Myc and cyclin D1, β-Catenin; ↓ invasion and metastasis;	[177]
	ZNF471 (zinc finger protein 471)	Cell differentiation, proliferation, apoptosis, neoplastic transformation, and metabolic pathway	Downregulated	↓ cell migration and invasion; ↑ cobblestone-like cells morphology and ↓spindle-like morphology; ↑ E-cadherin; ↓ vimentin, Snail, Slug, and N-cadherin; ↓ MMP1 and MMP3; ↓ expression of phospho-GSK-3β, active-β-catenin, and the downstream target genes: c-Myc, cyclin D1	[178]
	miR-429	Tumor suppressor	Downregulated	↓ invasion and migration abilities; ↓ vimentin; ↑ E-cadherin; ↓ F-actin accumulation in cytoplasm	[179]
	AQP5 (Aquaporin 5)	Facilitate the transport of water and other small solutes through the cell membrane	Overexpressed	↑ invasion and metastasis	[180]
	CDK14 (cyclin-dependent kinase 14)	Cell cycle regulation	Overexpressed	↑ invasion and metastasis	[181]
	DNER (delta/notch -like epidermal growth factor (EGF)-related receptor)	Neuron-specific transmembrane protein found in a variety of peripheral cells; mediates signaling through neuron–glia interactions	Overexpressed	↓ E-cadherin; ↑ N-cadherin, vimentin, Snail, β-catenin	[182]
	ENC1 (ectodermal-neural cortex 1)	Important role during early gastrulation and the formation of the nervous system	Overexpressed	↑ nucleus β-catenin expression; ↓ E-cadherin; ↑ N-cadherin and vimentin	[183]
	FNDC1 (fibronectin type III domain-containing protein 1)	A class of receptor-independent activators of G protein signal transduction, which can activate G protein signal by interacting with Gbγ subunit	Overexpressed	↓ E-cadherin; ↑ N-cadherin; ↑ EMT; ↑ β-catenin, c-Myc, cyclin D1, CDK4	[184]
	HePTP (hemato-poietic protein tyrosine phosphatase)	Inflammatory response and T-cell antigen receptor (TCR) signaling	Overexpressed	↑ cells mobility and invasiveness; ↑ GSK3β phosphorylation; ↑ nuclear β-catenin; ↑MMP7, SNAI2, MMP2, and CD44	[185]
	KRT6A (Keratin 6A)	Structural support and functional regulation of epithelial tissues such as skin, hair and nails	Overexpressed	↑ migratory abilities; ↑ CDH2, SNAIL, and vimentin; ↑ β-catenin stability; ↑ GSK3β phosphorylation	[186,187]
	KIF3B (kinesin family member 3B)	Regulator in mitotic progression	Overexpressed	↑ invasive ability; elongated morphological appearances and mesenchymal-like properties; ↓E-cadherin; ↑ vimentin, MMP-2, MMP-9, Slug and Snail; ↑ β-catenin	[188]
	KIF23 (kinesin family member 23)	Component of the centralspindlin complex and involved in the cytokinesis	Overexpressed	↑ cell migration and invasion; ↑ N-cadherin, vimentin; ↓ E-cadherin; ↑ β-catenin	[189]
	LGR4 (the leucine-rich repeat containing GPCR-4)	Tissue development and maintenance	Overexpressed	↓ E-cadherin; ↑ vimentin and ZEB1; ↑ EMT; spindle-shaped mesenchymal cell morphology; ↓ cortical F-actin	[190,191]
	LGR6	Stem cell marker in many tissues	Overexpressed	↑ metastasis abilities	[192]
	NUF2 (NDC80 kinetochore complex component)	Serves as a component of the NDC80 kinetochore complex and participates in binding of the kinetochore to microtubules during mitosis	Overexpressed	↑ MMP2, MMP7, SNAI1, SNAI2, and TWIST1	[193]
	PAX7 (Paired Box 7)	Nervous and muscular systems development	Overexpressed	↑ β-catenin, c-MYC, and cyclin D1; ↑ invasion and metastasis	[194]
	PGAM1 (phospho-glycerate mutase 1)	Glycolytic enzyme	Overexpressed	↑ GSK3β and β-catenin; ↑ invasion and metastasis	[195]
	PROX1 (prospero- homeobox protein 1)	Transcription factor that plays an important role in the formation of lymphatic vessels in animal embryo development	Overexpressed	Mesenchymal morphology predisposition; ↑ N-cadherin, vimentin, Slug, Twist2, ZEB1; ↓ E-cadherin; ↑ TCF4 LEF, Met and C-myc (important genes which are controlled by Wnt pathway)	[196]
	miR-374a	Regulatory role, such as in reproductive disorders, cell growth and differentiation, calcium handling in the kidney, various cancers and epilepsy	Overexpressed	↑ β-catenin; ↑ N-cadherin; ↓ E-cadherin; ↑ EMT	[197,198]
	SLC35A2 (Solute Carrier Family 35 Member A2)	Responsible for the transportation of nucleoside–sugar compounds	Overexpressed	↑ Twist1, Slug, N-cadherin, β-catenin and MMP-9; ↓ E-cadherin	[199]
	SALL4 (leucine-rich repeat-containing GPCR-6)	Zinc-finger transcription factor; Regulates pluripotency of embryonic stem	Overexpressed	↑ cell migration and invasion	[200]
	UBE3C (ubiquitin protein ligase E3C)	Regulator of proteasome function that continuously cycles on and off proteasomes and stimulates associations by ubiquitin conjugates through cooperation with USP14	Overexpressed	↑ invasion and metastasis; ↑ nuclear β-catenin	[201]
	UGCG (UDP-glucose ceramide glucosyltransferase)	Enzyme responsible for the production of glucosylceramide which is the precursor of all glycosphingolipids (GSLs) and essential for various cellular processes	Overexpressed	↑ vimentin, N-cadherin, and fibronectin; ↑ invasion and metastasis	[202]
	WHSC1 (Wolf–Hirschhorn syndrome candidate gene-1)	Important role in early growth and development; Histone methylation	Overexpressed	↓ E-cadherin; ↑ fibronectin and N-cadherin	[203]

Legend: ↑ activation or increase; ↓ inhibition or decrease.

**Table 5:** Molecules that regulate NF-κB signaling pathway in TNBC metastasis process.

Signaling Pathway	Upstream Regulator (Protein, RNA, Gene)	General Function	Expression in TNBC	Influence on Metastasis Formation in TNBC	References
NF-κB	Morgana/chp-1	Chaperone activity	Overexpressed	↑ MMP9; ↑ invasion and metastasis;	[208]
	DP103 (DEAD-box helicase)	Transcriptional repression and RNA helicase activity modulation	Overexpressed	↑ MMP9; ↑ branching morphogenesis; ↑ cell motility; ↑ p65 nuclear accumulation; ↑ ICAM-1 and CXCR4	[213,214]
	NTF4 (neurotrophin-4)	Development and maintenance of the nervous system, where they stimulate neuronal cell survival, differentiation, and plasticity	Overexpressed	↑ invasion and metastasis; ↓ E-cadherin; ↑ N-cadherin, SNAIL; ↑ EMT ↑ANXA1 and the phosphorylation of NF-κB; ↑ p-p65 nuclear localization	[215,216]
	PTPN20 (Protein Tyrosine Phosphatase Non-Receptor Type 20)	Cellular growth, proliferation, differentiation, migration and immune response	Overexpressed	↑ invasion and metastasis	[217]
	RBM7 (RNA Binding Motif Protein 7)	RNA surveillance activity as a component of the human nuclear exosome target (NEXT) complex	Downregulated	↓ invasion and metastasis; ↓ phosphorylation of p65	[218]
	TIPE3 (tumor necrosis factor–alpha-induced protein 8 (TNFAIP8)-like 3)	Apoptosis regulator	Overexpressed	↑ invasion and metastasis; ↑ IkBα and p65 phosphorylation; ↑ MMP2	[219]
	UGCG (UDP-glucose ceramide glucosyltransferase)	Enzyme responsible for the production of glucosylceramide which is the precursor of all glycosphingolipids (GSLs) and essential for various cellular processes	Overexpressed	↑ vimentin, N-cadherin, and fibronectin; ↑ invasion and metastasis	[102]

Legend: ↑ activation or increase; ↓ inhibition or decrease.

## Data Availability

No new data were created or analyzed in this study.

## Abbreviations

ADCs	antibody–drug conjugates
APC	adenomatous polyposis coli
BC	breast cancer
BL1	basal-like 1
BL2	basal-like 2
c-IAP	cellular inhibitor of apoptosis protein 1
CK1	casein kinase 1
CNS	central nervous system
COL10A1	collagen type X alpha 1
COL4A2	collagen type IV alpha 2
co-mediator SMADs	co-mediator SMADs
CSCs	cancer stem cells
CSF1	colony stimulating factor 1
CTCs	circulating tumor cells
CTSB	cathepsins B
CTSS	cathepsin S
ECM	extracellular matrix
EGF	epidermal growth factor
EMT	epithelial–mesenchymal transition
ER	estrogen receptor
FGF	fibroblast growth factor
FISH	fluorescence in situ hybridization
FN	fibronectin
GDF10	growth differentiation factor-10
GPCRs	G protein-coupled receptors
GSK3	glycogen synthase kinase 3
HA	hyaluronic acid
HDI	Human Development Index
HER2	human epidermal growth factor receptor 2
HGF	hepatocyte growth factor
HR	hormone receptor
ICAM-1	intercellular adhesion molecule-1
IHC	immunohistochemical
IKK	IκB kinase
IM	immunomodulatory
LAR	luminal androgen receptor
LOX	lysyl oxidase
LOXL4	lysyl oxidase-like 4
M	mesenchymal
MD	mammographic density
MIR	Mortality-to-Incidence Ratio
MLC	myosin light chain
MMPs	matrix metalloproteinases
MSL	mesenchymal stem-like
MYPT1	myosin phosphatase targeting subunit
NEMO/IKKγ	NF-κB essential modulator
NF-κB	nuclear factor-κB
NIK	NF-κB-inducing kinase
Par6	partitioning-defective protein 6
PDGF	platelet-derived growth factor
PD-L1	programmed cell death ligand 1
PI3K/mTOR	phosphoinositide 3-kinase/mechanistic target of rapamycin
PMN	pre-metastatic niche
PR	progesterone receptor
R-SMADs	receptor-regulated SMADs
RhoGAP	Rho GTPase-activating proteins
RhoGDI	Rho GDP dissociation inhibitors
RhoGEF	Rho guanine nucleotide exchange factors
ROCK1/2	Rho-associated coiled-coil-containing protein kinase 1 and 2
ROS	reactive oxygen species
RTKs	receptor tyrosine kinases
TAMs	tumor-associated macrophages
TCF/LEF	β-catenin-T-cell factor/lymphoid enhancer-binding factor
TGF-β	transforming growth factor-β
TILs	tumor-infiltrating lymphocytes
TIMPs	tissue inhibitors of metalloproteinases
TME	tumor microenvironment
TMEM	TME of metastasis
TNBC	triple-negative breast cancer
TNFR	TNF receptor
TβRI	type I receptor
TβRII	type II TGF-β receptor
VCAM-1	vascular cell adhesion molecule-1
VEGF	vascular endothelial growth factor
